# Exosomes from Adipose Tissue Mesenchymal Stem Cells, a Preliminary Study for In Vitro and In Vivo Application

**DOI:** 10.3390/bioengineering12101129

**Published:** 2025-10-21

**Authors:** Thao Duy Huynh, Ciro Gargiulo Isacco, Quan Thai Minh Ngo, Binh Thanh Nguyen, Tuan Ngoc Huu Nguyen, Tri Minh Dang Bui, Vinh Minh Ngo, Ky Quoc Truong, Tro Van Chau, Hoa Cong Truong, Kieu Diem Cao Nguyen, Emilio Jirillo, Van Hung Pham, Luigi Santacroce, Toai Cong Tran

**Affiliations:** 1 Biomedical Research Center, Pham Ngoc Thac University of Medicine, Ho Chi Minh City 700000, Vietnam; thao_huynhduy@pnt.edu.vn (T.D.H.); quanntm@pnt.edu.vn (Q.T.M.N.); binhnt.bmmophoi@pnt.edu.vn (B.T.N.); nhntuan@pnt.edu.vn (T.N.H.N.); tribdm@pnt.edu.vn (T.M.D.B.); vinhnm@pnt.edu.vn (V.M.N.); kytq@pnt.edu.vn (K.Q.T.); trocv@pnt.edu.vn (T.V.C.); hoatc@pnt.edu.vn (H.C.T.); 2 Interdisciplinary Department of Medicine, Section of Microbiology and Virology, School of Medicine, The University of Bari, 70124 Bari, Italy; emilio.jirillo@uniba.it (E.J.); luigi.santacroce@uniba.it (L.S.); 3 The Institute of Immunology, Gene, and Cell Therapy, Hanoi City 100000, Vietnam; drkieukaren@gmail.com; 4 Viet Nam Research and Development Institute of Clinical Microbiology, Ho Chi Minh City 756000, Vietnam; phhvan.nkbiotek@gmail.com

**Keywords:** adipose tissue mesenchymal stem cells (AT-MSCs), exosomes, miRNA, siRNA, H_2_O_2_-induced damage, UVB-induced damage, qRT-PCR

## Abstract

Mesenchymal stem cells (MSCs), particularly their secreted exosomes, small microvesicles, represent a major focus in regenerative medicine due to their therapeutic potential. Exosomes exhibit growth factors and cytokines and are loaded with microRNAs (miRNA) and short interfering RNA (siRNA) that can be transferred to other cells, potentially affecting their function. Exosomes are crucial mediators of intercellular communication, are immunomodulatory, and are promoters of tissue regeneration. Despite their promise, the standardized methods for exosome isolation and characterization remain weak. This exploratory study addresses this gap by detailing an effective method for isolating exosomes from adipose tissue mesenchymal stem cells (AT-MSCs), emphasizing precipitation as a technique yielding a high efficiency and purity compared to other methods. Functionally, we aimed to confirm the AT-MSC exosomes’ ability to exert an effective protective activity on the skin and its main components, such as fibroblasts, collagen, and elastin. To achieve this goal, we had to demonstrate that AT-MSC exosomes are safe and free of toxic substances. They can express specific proteins such as CD9, CD63, and CD81, which are well-known exosome markers. These exosomes also contain key miRNAs, including miRNA-203 A, miRNA-203 B, and miRNA-3196, important for skin regeneration, as well as enhancers of cell integrity and proliferation. We eventually confirmed the ability of exosomes to exert protective and recovery effects on fibroblasts after H_2_O_2_-induced damage in vitro, as well as on mouse skin after UVB-induced damage in vivo. These effects were verified by measuring levels of reactive oxidative species (ROS), assessing SA-β-Galactosidase (SA-β-Gal) activity, analyzing the cell cycle, evaluating the telomere length of fibroblasts by RT-PCR, and conducting histological assessments of collagen and elastin structure in murine skin after UVB exposure. This exploratory work provides valuable insights into the isolation, characterization, and bioactive and reparative properties of exosomes from AT-MSCs, supporting their development for future studies and therapeutic applications.

## 1. Introduction

By definition, stem cells are cells capable of self-renewal and differentiation into more specialized cells and tissues [1,2]. Recent advances in stem cell research have made stem cell-focused treatment a potential alternative for wound healing and tissue rejuvenation [3]. Currently, categorization models proposed for stem cells are based on (i) their source, such as bone marrow-derived MSCs (BM-MSCs), umbilical cord mesenchymal stem cells (UC-MSCs), hematopoietic stem cells (HSCs), placenta stem cells (PSCs), adipose tissue mesenchymal stem cells (AT-MSCs), and embryonic stem cells (ESCs); and (ii) their capacity of differentiation into other cells and tissues or “potency” ability, for which we have totipotent, pluripotent, and multipotent stem cells. Totipotent cells retain the ability to differentiate into any cell type in the body, including extraembryonic tissues. The pluripotent cells can differentiate into any cell type in the body, but not extraembryonic tissues, and multipotent cells can only differentiate into a limited number of tissues or cell lineages [4,5,6,7]. Pluripotent stem cells, such as hematopoietic stem cells found in bone marrow, can differentiate into various types of blood cells, while mesenchymal stem cells, found in bone marrow and other tissues such as the placenta, umbilical cord blood, adipose tissue, chorionic membrane, and amniotic fluid, can differentiate into bone, cartilage, muscle, neurological, and fat cells [8,9,10,11,12]. Either in vitro or in vivo, human AT-MSCs have shown remarkable plasticity, self-renewal, and immune modulation capacity, with a great ability of tissue repair [13,14,15]. Human AT-MSCs are easily harvested with a minimally invasive procedure and are easily cultured and grown in vitro with a higher MSC yield [16]. Compared to other types of MSCs, AT-MSCs have been shown to have similar biological features and a long lifespan, high proliferative capacity, short ploidy time, and late senescence rate, making them ideal for cell therapy for chronic and persistent diseases [17,18]. In general, AT-MSCs and their derived exosomes are characterized using a multi-analysis approach based on morphology, as well as the expression of stem cell markers, which is confirmed by flow cytometry and fluorescence analysis, RT-PCR, and SEM (scanning electron microscopy).

## 2. Aging Intended as Disease, a Different Epistemological Perspective

The current paradigm views aging as an inevitable biological process that involves a series of structural and functional changes, mainly due to the constant decline of cells, tissues, and organs [19]. However, this concept is not exhaustive and is often misleading. It requires an epistemological approach in the way one should consider aging as a disease. Research has highlighted the presence of several “hallmarks” of aging, including genomic instability, telomere shortening, and mitochondrial dysfunction, which characterize age-related decline—a gradual deterioration of physiological integrity, making individuals more susceptible to disease and mortality. This view opens to different medical and clinical perspectives [20]. Accordingly to recent data, 23% of people between the age of 50 to 65 have at least three long term pathologies; 36% of males over the age of 80 need support for their daily routines, while 49% of women over the age of 80 need care support in their daily routine. Modulating aging will consequently have several impacts on diseases and could extend people’s health span [21]. In this regard, model studies have highlighted the presence of molecular stains on aged cells that become targets for new drugs capable of prolonging their life [22]. Furthermore, while the genes responsible for longevity have been highlighted, more attention is being given to healthier daily activities such as physical exercise and intermittent fasting, especially regarding their effect on telomeres and telomerase [22,23,24].

### 2.1. Senescence Process and Related Pathological Mechanisms

Telomere length and DNA damage response are deeply interconnected in the regulation of cellular aging. Aging is characterized by telomere shortening and restless states of inflammation, which in turn promote senescence through the constant accumulation of proinflammatory factors [24]. Proinflammatory leukocytes, cytokines such as IFN-γ, IL-6, and TNF-α, and increased levels of nuclear factor kappa-beta (NF-kB) have been linked with short leukocyte telomeres, an increased cellular oxidation rate, and high reactive oxygen species (ROS) in cells and tissues [23,24]. The accumulation of senescent cells, premature shortening of telomeres, or unexplained lengthening of telomeres are all signs of the aging process, which occurs secondary to cellular damage due to biological and environmental stress, characterized by defective repair [25,26]. In this context, the role of stem cells is important. It is assumed that induced tissue damage and the proliferation of damaged tissue-specific stem cells promote accelerated telomere shortening, also in response to increased intrinsic inflammatory factors [27]. These cells undergo continuous cycles of damage and repair, driven by the differentiation of mature stem cells [27,28]. Damaged telomeres promote the dysfunction of cellular organelles, such as mitochondria, reducing their ability to scavenge ROS. Based on the Hayflick limit, where cells have a finite number of divisions, telomere shortening becomes a clear sign of aging, closely associated with diminished cell production and regeneration [28,29]. Studies have shown that either uncontrolled longer or shorter telomeres show premature aging and reduced longevity [30,31].

The nucleus is the most affected site during the aging process, as it is involved in the entire cell cycle, telomere activity, and epigenomic changes, and plays a role in reducing the integrity of nuclear DNA (nDNA) [32,33]. There is a bidirectional communication pathway between mitochondria and the nucleus that can also impact nDNA. This relationship is mediated by the mitochondrial translocation of human telomerase reverse transcriptase (hTERT) from the nucleus, where it binds to mitochondrial DNA (mtDNA) and mitochondrial tRNA [34,35,36]. Interestingly, this pathway has been shown to enhance mitochondrial protection following increased oxidative stress (OS), as hTERT increases the mitochondrial membrane’s potential to reduce ROS and protect mtDNA [34,35,36]. Communication with the nucleus indicates the oxidative status of mitochondria through the translocation of the telomerase RNA component of hTERC [34,35,36]. In addition, it was seen that hTERT can be upregulated in cells with rapid division, including embryonic stem cells and adult stem cells, where it helps maintain telomere length [34,35,36]. Degenerative patterns affecting the nucleus have been observed both directly and indirectly in the cytoplasmic and extracellular matrix, where signaling pathways related to inflammation and fibrosis are primarily expressed [35]. Scientists have identified many proinflammatory factors and chemokines that contribute to the increase in senescent cells, which, in turn, trigger complementary aging signals that induce the transformation of surrounding healthy cells [35,36].

Mitochondrial oxidative metabolism is upregulated in senescent cells to satisfy a simple metabolic need [37,38]. Of note, cell cycle arrest in senescence is largely mediated by the activation of one or both of the p53/p21^WAF1/CIP1^ and p16^INK4A/pRB^ tumor suppressor pathways, which have a strict connection with mitochondrial activity. The generation and accumulation of free radicals are mainly associated with the gradual decline of mitochondrial function in aging cells, attributed to the overproduction of ROS [37]. The mitochondria of skeletal muscle tissue cells of older animals showed that both mitochondrial bioenergetics and membrane potential differences were significantly impaired compared to those from younger animals. The oxidative damage of transcribed proteins and mtDNA is associated with the accumulation of mtDNA mutations. This raises the question of whether aging, when accompanied by significant mitochondrial and other disease-causing cellular dysfunction, should itself be considered a disease [37,38].

### 2.2. In Vitro Experiments to Induce Aging

Both in vitro and in vivo results obtained from artificially induced aging models using H_2_O_2_ and UVB exposed the important role of miRNA and telomeres in different cellular aging pathways. The results demonstrated a crucial link between the various components underlying the aging process: telomeres, miRNAs, p53, and p21. For example, telomeres, which protect the ends of chromosomes, are linked to this pathway, as telomere shortening tends to trigger p53 activation and subsequent cell cycle arrest or senescence. Furthermore, microRNAs (miRNAs) are involved in fine-tuning the p53/p21 pathway: some miRNAs directly regulate the expression of p53 or p21, while others influence telomere maintenance [39,40,41,42].

#### 2.2.1. The Use of H_2_O_2_ and SA-Β-Galactosidase to Induce Senescence In Vitro

In vitro exposure to H_2_O_2_ and SA-β-Galactosidase (SA-β-Gal) has been shown to induce cellular senescence and modulate the expression of miRNAs and telomeres. Specifically, H_2_O_2_ can induce oxidative stress and cellular senescence in fibroblasts, thereby affecting telomere length and miRNA expression, potentially contributing to cellular aging and disease, which in turn influences mitochondrial function through specific signaling pathways, particularly the p53-peroxisome proliferator-activated receptor gamma 1α (PGC-1α) pathway [41,42,43]. Contrary to what was previously believed, mitochondrial H_2_O_2_ release and ROS production affect nDNA and telomeres through sophisticated signaling mechanisms rather than through direct oxidative damage [44,45,46]. Accumulating evidence indicates that some members of the H_2_O_2_ reductase family play a role in regulating the redox state of telomerase thiols, which in turn influences cellular redox homeostasis [47]. These reductases, including peroxiredoxins and glutathione peroxidase, use H_2_O_2_ to modulate thiol modifications on proteins, including miRNAs within exosomes, thereby influencing their activity at telomeres and cellular redox balance [48,49,50,51]. The SA-β-gal enzyme is a distinctive marker of cellular senescence. It is highly expressed at pH 6.0 in senescent cells, with increased activity in response to stressors such as oxidative stress, DNA damage, or prolonged cell division (replicative senescence) [52]. SA-β-gal activity is widely considered a reference biomarker for assessing cellular aging. In vitro experiments using SA-β-gal as an inducer of fibroblasts’ senescence and pathophysiological processes related to aging were also performed specifically to target p53, known to have multiple effects on gene expression and on senescence, determined by the transcriptional activation of p16^INK4A^ and p21^Wai1/CiP1/Sdi1^, both crucial in DNA repairing while inhibiting apoptosis [52,53,54].

Intracellular ROS levels can be assessed by co-staining with Hoechst 33342 and DCFH-DA, followed by fluorescence imaging and image analysis [55]. The results confirmed that the activation of p53, p21, and p16 was only expressed in those senescent cells during the transient process; eventually, these protein levels decreased after growth arrest occurred. Mechanistically, DCFH-DA serves as an intracellular marker. Upon crossing the cell membrane, its acetyl groups are cleaved by esterase enzymes, yielding DCFH [56]. Subsequently, intracellular ROS oxidizes DCFH to form DCF, a fluorescent marker emitting green fluorescence within the emission spectrum [56]. Following a series of intracellular reactions, relative ROS levels can be assessed based on the fluorescence intensity of the reaction products. ROS levels can also be evaluated by determining the number of viable cells at the experimental time point, with cell nuclei stained using Hoechst 33342 for quantification [57,58,59].

#### 2.2.2. The Use of UVB to Induce Senescence In Vivo

Several other elements may provoke aging. Externally induced aging is caused by cumulative exposure to environmental stimuli, such as ultraviolet radiation, environmental toxins, chemicals, and infectious agents, which lead to DNA alterations and skin damage [60,61,62]. UV rays from sunlight are the most damaging external component to the skin; therefore, extrinsic aging is also called photoaging. UV rays penetrate the skin and interact with different cells, inducing distinct, yet overlapping, biological responses in both epidermal and dermal structures [63]. There is a growing recognition of the role of UVB in increasing ROS and the subsequent damage to nuclear and mitochondrial DNA, telomeres, cell membrane lipids, or proteins [63]. Data have shown that ROS becomes detectable immediately after UVB exposure, with a peak within approximately 30 min. Repeated exposure contributed to the excessive consumption of antioxidant factors, which led to the accumulation of OS, responsible for premature aging or cell death through the activation of proliferation and survival signaling pathways, including MAP kinase, PI3 kinase, PTEN, and protein tyrosine phosphatase [62,63]. Furthermore, the vast majority of cellular ROS (about 90%) can be traced to mitochondrion, which is also susceptible to ROS attack [64,65].

The main intent of this study was to determine and characterize exosomes obtained from human AT-MSCs that may represent a valid opportunity in the field of regenerative and antiaging medicine. After a general introduction, the therapeutic, regenerative, and applicative roles of AT-MSC exosomes are discussed in their respective sections. In this study, we cultured AT-MSCs, extracted exosomes from their supernatants, and collected and isolated fibroblasts. In in vitro experiments with fibroblasts and in vivo experiments with mice models, we found that AT-MSC exosomes significantly promoted healing from SA-β-galactosidase-, H_2_O_2_-, and UVB-induced damage. The results showed that the exosomes exerted a dual function of protection and regeneration against increased levels of oxidative stress, ROS, and apoptosis. More importantly, the exosomes could significantly modify cellular and tissue damage through the presence of the specific miRNAs (203 A and B and 3196) that were detected in the study.

## 3. Material and Methods, Experimental Design, and Statistical Analysis

All procedures were performed under informed consent, following human study protocols approved by the Declaration of Helsinki for the reuse of human and animal biospecimens in scientific research and the Biosolution of the Vietnam National Institute of Health (IRB number: 1039/TDHYKPNT-HHDD signed 24 January 2024; Research n93IRB-VN01013; grant number B2019-44-01).

### 3.1. In Vitro Fibroblast Isolation

Fibroblasts were collected from fresh skin biopsy (1 cm^2^) of 10 healthy male donors, aged 40–45 years (tested with different panels for infectious agents such as HIV, HBV, HCV, and VDRL), isolated and processed in the laboratories of Pham Ngoc Thach University, and transported in “blank” DMEM (Merck (Rahway, NJ, USA), Sigma-Aldrich (St. Louis, MO, USA)). The fibroblasts were obtained by a mechanical dissociation in a polystyrene Petri dish. Here the tissues were incised 10–12 times for softening; 5 mL PBS-A was then added to the incised tissue, and it was left at room temperature for 5 min. hFs were proliferated in T-25 culture flasks using DMEM/F-12 medium supplemented with 10% fetal bovine serum (FBS) and 1% Penicillin–Streptomycin. Upon reaching confluence, hFs were detached from the flasks using trypsin-EDTA. We removed the tissue from the PBS-A and we transferred the sample into an enzymatic procedure in a 50 mL Falcon tube with 100 μL Collagenase P and 40 μL DNase stock solution to 5 mL of “complete” DMEM. We placed the 50 mL Falcon tube in the orbital shaker, and then we incubated it at 37 °C with agitation (200 rpm) for 15 min. After 15 min, it was removed from the orbital shaker and sequentially pipetted with 50 mL, 10 mL, and 5 mL pipette tips to promote dissociation with trypsin-EDTA. The sample was then returned to the orbital shaker for a further 15 min, and then sequential pipetting was repeated. The sample was returned to the orbital shaker for a further 30 min, then sequential pipetting was repeated. The sample was centrifuged at 450× *g* for 5 min. Then, we removed the supernatant and resuspended the resulting pellet in 1 mL of undiluted TrypLE. Then, we incubated it at 37 °C for 10 min, and non-digested tissue fragments were manually removed. We added a tissue strainer to a new 50 mL Falcon tube on ice. Tissue/enzyme suspension was strained and pressed through with the plunger of a 5 mL syringe, while simultaneously washing with “empty” DMEM. At this time, we kept the sample on ice. The sample was centrifuged at 450× *g* for 5 min at 4 °C. We then aspirated the medium and resuspended the pellet in 1 mL red blood cell lysis solution. The sample was incubated at 4 °C for 10 min. We centrifuged it at 450× *g* for 5 min at 4 °C; we collected the red cell lysis buffer. We resuspended the pellet in 1 mL of “complete” DMEM.

### 3.2. The AT-MSC Isolation Procedure and Identification

Adipose tissue was collected from liposuction at the General Hospital of Ho Chi Minh City, Vietnam. Fat tissues were originally collected from consenting healthy donors (males and females between the ages of 45 and 50 years old and tested with different panels for infectious agents such as HIV, HBV, HCV, and VDRL). Abdominal fat was harvested endoscopically in a lipoaspirate form and transferred to a laboratory facility for further processing. Samples were collected in a sterile syringe or tubes, put into a refrigerated container, quickly delivered to our laboratory, and stored at 2–8 °C. The samples were processed in sterile conditions and transferred in a conical tube of 50 mL (Mercks, Hamburg, Germany); collagenase type I and dipase were added (from Gibco, Paisley, UK) and the solution was shaken gently and successively incubated at 37 °C for 90 min. During incubation, the samples were shaken every 15 min; after 90 min, the samples were centrifuged at 3000 RPM for 5 min at 4 °C. The supernatant fat and liquid were removed, leaving the pellet untouched at the bottom of the tube; DMEM/F-12 medium containing 10% FBS (Gibco, Paisley, UK) and 1% Penicillin/Streptomycin (*v*/*v*) was added, and cells were suspended with a pipette in the medium. We attached a 70 μm cell strainer to the sterile conical tube and we filtered the cell suspension into the new tube. The tube was centrifuged at 3000 RPM for 5 min at 4 °C; we discarded the supernatant carefully without touching the pellet. We resuspended the pellet in serum and antibiotic-containing culture medium. Cells were counted and were assessed for viability by using an automated cell counter (Beckman Coulter/Vi-CELL XR, Indianapolis, IN, USA); cells were cultured into in T25/T75 flasks (Thermo-Fisher, Jangsu, China) and put into a cell incubator at 37 °C, 5% CO_2_ (LEEC.Touch 190S-T, Nottingham, UK). AT-MSCs were isolated through an enzymatic process from adipose tissues collected from consenting donors and tested with different panels for infectious agents such as HIV, HBV, HCV, and VDRL. Flow cytometry analysis confirmed the presence of MSC typical markers. Results of the evaluation with flow cytometry were used to identify AT-MSCs CD-44, CD-73, CD-90, CD-19, and CD14low; the results were negative for CD45 and HLA-DR.

### 3.3. The AT-MSC-Derived Exosome Profile and Characterization

At passage n.2, we removed the complete cell culture medium once hAD-MSCs reached about 70–80% confluency in cell culture flasks. A serum-free StemPro MSC SFM (Gibco, Norristown, PA, USA) was used to culture MSCs from adipose tissue for 48–72 h. In biological safety cabinet class II (Keuwanee, Bengaluru, India), with a pipette (Eppendorf, Hamburg, Germany), the culture media was transferred to 50 mL sterile conical tubes (Biologix, Camarillo, CA, USA) and centrifuged at 300× *g* for 10 min at 4 °C using the cooling UNIVERSAL 320R centrifuge (Hettich, Kirchlengern, Germany). In the second step, we collected the supernatant and transferred it to the new conical tubes to be centrifuged at 2000× *g* for 10 min at 4 °C; the supernatant was transferred to sterile ultracentrifuge tubes (Beckman Coulter, Indianapolis, IN, USA), sealed with titanium caps (Beckman Coulter, USA), and centrifuged in an Optima XPN-90 ultracentrifuge (Beckman Coulter, USA) at 10,000× *g* for 10 min at 4 °C. The supernatant was collected and transferred to the new ultracentrifuge tubes and centrifuged at 100,000× *g* for 70 min at 4 °C. The exosomal pellets located at the bottom of the tubes were collected after discarding the supernatant carefully. PBS 1× (Gibco, USA) was added to remove unwanted components and centrifuged at 100,000× *g* for 70 min at 4 °C. After ultracentrifugation, the tubes were moved into biological safety cabinet class II to discard the supernatant carefully, and exosomes were resuspended with 1 mL PBS 1X. Eight tubes of exosomes, each with a volume of 1.0 mL, were obtained via ultracentrifugation for the subsequent analysis and evaluation of exosomes in further experiments. The average size of vesicles in the exosome suspension, ranging from 40 to 154.50 nm (as assessed by FE-SEM and DLS procedures), serves as evidence that the exosomes in the sample meet ISEV size standards for potential applications. The vesicle sizes are aligned with earlier studies, confirming the sample’s stability and its potential for therapeutic applications, such as contrasting cellular aging in fibroblast models, as particles of this size are readily absorbed by fibroblasts. Flow cytometry analysis confirmed the presence of exosomes, being positive for CD9, CD63, and CD81 markers, which are highly characteristic proteins for exosome identification. In addition, exosomes were checked for the presence of miRNA, and more specifically miRNA 203A, miRNA 203B, and miRNA 3196, known to modulate various cellular processes, including cell growth, metabolism, and regenerative and immune responses to stimuli. The exosomes were collected with a sterile syringe (VINAHANKOOK, Hanoi, Vietnam), poured into a special container, and stored at −20 °C in a freezer (Liebherr, Bulle, Switzerland) no longer than 6 months.

### 3.4. In Vitro Exposure of hF to H_2_O_2_ and Experimental Design of Exosomes to Assess ROS Levels

hFs were seeded into 96-well plates at a density of 10^4^ cells per well. After 24 h of incubation, experimental groups were established with three replicates per group: positive control, negative control, and study groups with exomes named, respectively, EXO-1, EXO-5, EXO-10, EXO-15, and EXO-20. Experiment 1—Control: No exposure to exosomes and no treatment with H_2_O_2_; telomere length resulted. Experiment 2—Fibroblasts were exposed to exosomes (5 µg/mL) for 24 h, followed by exposure to 200 µM H_2_O_2_ for 90 min. Experiment 3—Fibroblasts were exposed to 200 µM H_2_O_2_ for 90 min, followed by exposure to exosomes (5 µg/mL) for 24 h. Experiment 4—Fibroblasts were exposed to exosomes only for 24 h. Experiment 5—Fibroblasts were exposed to 200 µM H_2_O_2_ for 90 min. After 24 h of hF seeding in the 96-well plate, the medium was removed from all wells and 100 µL of Hoechst 33342 was added to each well. Each plate was incubated at 37 °C with 5% CO_2_ for 10 min; after incubation, the solution was removed, and the wells were washed with sterile 1X PBS. Then, 100 µL of DCFH-DA was added to each well. The 96-well plates were then incubated at 37 °C with 5% CO_2_ for 30 min. After incubation, the solution was removed, and the wells were washed with complete DMEM/F-12 medium. The wells were washed again with sterile 1X PBS, and fluorescence confocal imaging was performed using a cell screening system designed for research purposes:

+ Images of nine regions (positions within each well) per well were acquired.
+ Hoechst 33342 signals were recorded with an excitation wavelength of 355–385 nm and an emission wavelength of 430–500 nm.
+ DCFH-DA signals were recorded with an excitation wavelength of 460–490 nm and an emission wavelength of 500–550 nm.
+ Cell count and fluorescence intensity per image were analyzed using the Harmony software Version 3.0 integrated into the imaging system.

The ratio of DCF fluorescence (dichlorofluorescein) intensity to hF cell count is directly proportional to intracellular ROS levels in hFs. A higher ratio of DCF fluorescence intensity to hF cell count indicates elevated ROS levels in hFs. A lower ratio of DCF fluorescence intensity to hF cell count indicates reduced ROS levels in hFs.

### 3.5. In Vitro Assessment of Senescence-Associated β-Galactosidase Activity in hF After Exosome and H_2_O_2_ Exposure

β-Galactosidase is a hydrolytic enzyme present in the lysosomes of cells. In normal cells, the lysosomal pH is approximately 4. In contrast, during cellular aging, β-galactosidase activity increases at a lysosomal pH of 6, with this highly active form identified as senescence-associated β-galactosidase (SABG). SABG elevated enzymatic activity at pH 6 has been reported to coincide with the expression of cell cycle inhibitory genes and organelle damage due to increased intracellular ROS. In biomedical research, SABG is widely proposed as a common marker for detecting cellular aging. Histochemical staining with 5-bromo-4-chloro-3-indolyl-β-D-galactopyranoside (X-gal) as a substrate is recommended as a rapid and straightforward method to assess SABG expression. The procedure for evaluating cellular aging in hFs was executed and adapted to the scope following the original protocol of a senescence cell histochemical staining kit (Code: CS0030, Sigma, Hambourg, Germany) to suit experimental conditions. After hF exposure to exosomes and H_2_O_2_ treatment, cells from a 6-well plate were seeded into a sterile 24-well plate at a density of 3 × 10^4^ cells per well. Experimental groups were arranged in the 24-well plate, corresponding to groups from the 6-well plate, with three replicates per group (3 wells). Groups included negative control, positive control, EXO-0.1, EXO-0.5, EXO-1, and EXO-5. After 48 h of seeding hFs into the 24-well plate, the senescence cell histochemical staining kit was used according to the accompanying protocol, with adjustments for experimental conditions. The medium was removed from all wells. Cells in each well were washed twice with 200 µL of 1× PBS per wash. In total, 300 µL of 1× fixation buffer was added to each well, and the plate was incubated at room temperature for 7 min. Cells in each well were washed three times with 200 µL of 1× PBS per wash. A total of 200 µL of 1× staining mixture containing X-gal was added to each well. The plate was incubated overnight.

Following incubation, color imaging of cells in each well was performed using an inverted microscope equipped with a camera:

+ Three random regions per well were shot.
+ The number of blue-stained cells was counted based on the recorded images.
+ The total number of cells (blue-stained and unstained) was counted based on the recorded images.

hFs exhibiting blue staining were identified as senescent cells. The ratio of blue-stained hFs to the total hF count reflects the degree of cellular aging:

+ A higher ratio of blue-stained hFs to total hFs indicates a greater degree of cellular aging.
+ A lower ratio of blue-stained hFs to total hFs indicates less cellular aging.

### 3.6. Assessment of hF Cell Cycle in Asynchronous Cultures Following Treatment with Exosomes and H_2_O_2_ Exposure

The percentage of hFs in the G1, S, and G2 phases of the cell cycle was found. The aim of this section was to figure out the cell cycle status of hFs following exposure to exosomes and H_2_O_2_ treatment. Cell cycle analysis offers critical insights into the protective and regenerative abilities of exosomes under H_2_O_2_-induced oxidative stress. After hF exposure to exosomes and H_2_O_2_ treatment, cells from a 6-well plate were suspended in 1.5 mL centrifuge tubes with a minimum of 10^4^ cells per tube. Tubes were arranged to stand for experimental groups corresponding to those in the 6-well plate. Groups included negative control, positive control, EXO-0.1, EXO-0.5, EXO-1, and EXO-5. An amount of 1 mL of sterile 1× PBS was added to each tube, and cells were evenly suspended. After centrifugation, the supernatant was removed from each tube. Next, 70% ethanol was added dropwise to each tube until the volume reached 1 mL, with vortexing during addition. Tubes were incubated overnight. Following incubation, tubes were centrifuged at 3000 RPM for 10 min at 4 °C. After centrifugation, the supernatant was removed from each tube. A total of 500 µL of sterile 1X PBS was added to each tube, and cells were evenly suspended. Tubes were centrifuged at 3000 RPM for 10 min at 4 °C. After centrifugation, the supernatant was removed from each tube. In total, 50 µL of RNase (100 µg/mL) was added to each tube and incubated at room temperature for 30 min, protected from light. Following RNase incubation, 500 µL of PI/Triton X-100 (1 µg/mL) was added to each tube. Tubes were incubated at 4 °C for 30 min, protected from light. Samples were analyzed using a cell analysis system. Results were collected and visualized using the Kaluza software Version 3.0 installed on the cell analysis system’s computer.

### 3.7. Evaluation of hF Proliferation and Toxicity Following Treatment with Exosomes via the MTT Assay

The objective of this section was to investigate whether exosomes at varying protein concentrations can protect hF and promote normal proliferation following H_2_O_2_-induced damage. The MTT (3-(4,5-dimethylthiazol-2-yl)-2,5-diphenyltetrazolium bromide) proliferation assay relies on the reduction of MTT to formazan crystals catalyzed by dehydrogenases, enzymes derived from the metabolic activity of living cells. An increase in viable cell numbers correlates with an elevated dehydrogenase activity in the sample, allowing direct quantification of dissolved formazan via optical density measurements to assess cell proliferation. The procedure for evaluating cell proliferation in hFs was performed after hF exposure to exosomes and H_2_O_2_ treatment; cells from a 6-well plate were seeded into a sterile 96-well plate at a density of 10^3^ cells per well. The 96-well plate had experimental groups that were the same as the 6-well plate groups. There were groups for negative control, positive control, EXO-0.1, EXO-0.5, EXO-1, and EXO-5. We looked at proliferation on days 1, 5, and 9, with three copies of each group and time point. After 24 h of seeding hFs into the 96-well plate, the medium was removed from all wells on the designated day for each experimental group. day 1 wells were washed with sterile 1X PBS. In total, 90 µL of DMEM/F-12 medium and 10 µL of MTT were added to day 1 wells. The 96-well plate was incubated at 37 °C with 5% CO_2_ for 3 h. Following incubation, the solution was removed from day 1 wells. A total of 100 µL of 100% DMSO was added to each day 1 well, gently shaken, and incubated at 37 °C for 30 min. A new 96-well plate was prepared. After incubation, the solution from each day 1 well was transferred to the corresponding well in the new 96-well plate. Optical density was measured at 570 nm using a multi-well plate spectrophotometer. Proliferation assays for day 5 and day 9 were performed following the same steps as for day 1 (as described above). For wells awaiting evaluation at later time points, hFs were cultured in DMEM/F-12 medium supplemented with 10% FBS and 1% Penicillin–Streptomycin antibiotic. A higher optical density value at 570 nm indicates a greater number of viable cells.

### 3.8. Assessment of Exosome Cytotoxicity on Fibroblasts In Vitro

The cytotoxicity assessment was conducted using the MTT assay, in accordance with ISO-10993-5 [66]. Dead cells or those lacking enzyme activity did not produce formazan. Since the amount of formazan is directly proportional to the number of viable cells, optical density measurements at 570 nm following MTT treatment can be used to assess the metabolic activity of living cells. Evaluating the cytotoxicity of exosomes on fibroblasts in vitro was a critical step in exploring the application of exosomes in regenerative medicine, cancer therapy, and other therapeutic modalities. The main objective of this section was to determine the safety grade of exosome concentrations for their use in in vitro experiments with hFs and possible future application in vivo. Exosomes may contain potentially harmful components such as proteins, RNAs, or adverse lipids. Therefore, the in vitro experiments on hFs enabled us to assess whether exosomes induce cellular damage through apoptosis or necrosis; to identify the maximum exosome concentration that cells can tolerate without adverse effects, thereby informing the design of treatment protocols or appropriate usage guidelines; and to ensure that exosomes do not induce oxidative stress or inflammatory responses, which is particularly critical for therapeutic or cosmetic applications. In addition, evaluating the impact of exosomes on fibroblasts facilitates the understanding of biological interactions, since exosomes may either stimulate or inhibit cellular signaling pathways, influencing fibroblast proliferation, differentiation, and metabolism. Through experiments measuring reactive oxygen species (ROS), gene expression, or cellular stress analysis, researchers can uncover how exosomes modulate biological processes.

According to ISO-10993-5, the cytotoxic potential of a test substance is reflected by cell viability. A cell viability below 70% indicates that the test substance is cytotoxic to cells. Cell viability was calculated using the following formula:

Cell Viability (%) = (OD570T/OD570C) × 100

where OD570T is the optical density at 570 nm of the experimental group wells and OD570C is the optical density at 570 nm of the negative control group wells.

### 3.9. The Use of Exosomes In Vivo with Albino Mice Experimental Design

The in vivo experiment was conducted on Swiss Albino mice (males) exposed to medium-wave ultraviolet radiation (UVB, 280–320 nm) for 3 h, and exosomes were applied on dorsal skin (hair removed, 2 × 2 cm area). Three groups of mice were used: (1) negative control (n = 7; two mice were sacrificed every 2 weeks; one mouse for backup, used in week 6), with no exosome exposure and no UVB irradiation; (2) positive control (n = 7; two mice were sacrificed every 2 weeks; one mouse for backup, used in week 6), with no exosome exposure and only UVB irradiation; and (3) EXO-50 study group (n = 7; two mice were sacrificed every 2 weeks; one mouse for backup, used in week 6), with each mouse receiving 20 μL at 50 μL/mL on shaved dorsal skin (2 × 2 cm) after 3 h of UVB irradiation. Visual assessment was performed with a digital camera at the time points of week 2, week 4, and week 6 for the three groups. For each group, histological assessment was performed at week 2, week 4, and week 6, with HE staining and Trichrome staining; two mice were used at each time. We followed the ISEV (International Society for Extracellular Vesicles) guidelines for the identification and characterization of exosomes, also known as extracellular vesicles (EVs), published in the “MISEV2018” document (Minimal Information for Studies of Extracellular Vesicles), which is considered the gold standard in exosome research.

### 3.10. Dynamic Scattering Technique (DLS)

DLS was used as a standard procedure to assess the size of exosomes and their distribution. A narrow distribution curve indicates sample homogeneity, reflecting high exosome quality. DLS uses a laser to illuminate a sample containing nanoscale particles in a liquid suspension. The particles move randomly due to Brownian motion. The intensity of the scattered light fluctuates depending on particle movements, the procedure allows the determination of the particle size distribution.

### 3.11. Telomere Length Experimental Design

This experiment aimed to elucidate the role of exosomes in protecting and regenerating telomeres, thereby providing data to support applications in regenerative medicine and anti-aging therapies. Measurement of telomere length in the hF model was performed by using real-time PCR. Based on the findings from Section 3.4 and Section 3.5, we identified an optimal exosome concentration with effective protective effects on hFs, which will be utilized in the subsequent experiment. The optimal exosome concentration was established at A µg/mL and successively employed in the following experiment. This method relies on calculating the ratio of telomere-containing DNA (T) to a single-copy reference gene (S) within the genome. The T/S ratio (telomere-to-single-copy gene ratio) reflects the average telomere length in the cells and is commonly used as a quantitative measure in telomere length analysis by real-time PCR.

### 3.12. Human Fibroblast Telomere Measurement qPCR

The hFs were cultured in 35 mm dishes at a density of 5 × 10^5^ cells. Once the cells reached 70–80% confluence on the dish surface, they were subjected to the experimental conditions explained above. Human fibroblasts were collected from well culture sites, transferred to a fresh tube using a Pasteur pipette, diluted with 3× volume of HBSS at room temperature, and then centrifuged at 180× *g* for 10 min. After discarding the supernatant, the cell pellet was resuspended in HBSS. Cell counting and viability assays were performed. Total DNA from hFs was extracted using the KingFisher Flex system (Thermo Fisher Scientific, Walthem, MA, USA). Primer pairs for telomeres were designed to amplify the TTAGGG repeat sequences (primer pair for the reference gene: ID 280824-240819203). The qPCR thermal cycling setup used to assess telomere length consisted of an initial enzyme activation step at 95 °C for 10 min, followed by 40 cycles of denaturation at 95 °C for 15 s and annealing/extension at 60 °C for 1 min. Reference Gene PCR: The thermal program was similar but optimized for the specific primer pair. The evaluation method consisted of recording the cycle threshold (Ct) values for the telomere (T) and reference gene (S). Then, the T/S ratio was calculated, comparing the T/S ratios between sample groups to the lower relative telomere length. The real-time qPCR method described by O’Callaghan and Fenech was used based on the Cawthon procedure for relative telomere length (TL) measurement with a modification to use an oligomeric standard to quantify TL. Power SYBR I Master Mix (Applied Biosystems, Walthem, MA, USA, #4367396) contains AmpliTaq Gold DNA polymerase, dNTPs, SYBR I Green Dye, optimized buffers, and passive reference dye (ROX). For this study, the β-globin gene (hgb) was used as a single copy reference. Kinetic real-time quantitative PCR determines the number of fractional cycles (Ct) at which the accumulated fluorescence of the well exceeds a set threshold of 40, which is several standard deviations above the background fluorescence. A standard curve is established by diluting known amounts of a synthesized 84-meroligonucleotide containing only TTAGGG repeats. The number of repeats in each standard is calculated using standard techniques; the oligomer standard is 84 bp long (TTAGGG repeated 14 times), with a molecular weight (MW) of 26,667.2; the weight of the telomere standard is 2.6667 × 10^4^/6.02 × 10^23^ = 0.44 × 10^−19^ g. The highest concentration standard (TEL STD A) has 60 pg of telomeric oligomer (60 × 10^−12^ g) per reaction, so there are 60 × 10^−12^/0.44 × 10^−19^ = 1.36 × 10^9^ oligomer molecules in TEL STD A. The quantity of telomeric sequence in TEL STD A was determined using the formula: 1.36 × 10^9^ × 84 (oligomer length), resulting in 1.18 × 10^8^ kilobases of telomeric sequence. A standard curve was generated using absolute telomere length quantitative polymerase chain reaction (aTL qPCR) assay calculations for serial dilutions of TEL STD A, ranging from 10^−1^ (1.18 × 10^8^) to 10^−6^ (1.18 × 10^3^). To maintain a constant 20 ng of total DNA per reaction tube, plasmid DNA (pBR322) was integrated into each standard. Telomere sequence information per sample in kb was measured using the standard curve. The evaluation method was as follows: (i) record the cycle threshold (Ct) values for the telomere (T) and reference gene (S); (ii) calculate the T/S ratio as follows:

*T*/*S* = 2^*Ct*^*gene*−*^Ct telomere^*

Compare the T/S ratios between sample groups to the lower relative telomere length.

### 3.13. Exosome Protein Quantification

Samples were diluted with PBS to avoid exceeding levels of proteins that may disturb the detection of the Bradford method. We prepared a BSA standard solution at different concentrations, e.g., 0 µg/mL, 2 µg/mL, 4 µg/mL, 8 µg/mL, 16 µg/mL, and 32 µg/mL. We prepared the Bradford reagent (Coomassie Brilliant Blue G-250) according to the manufacturer’s instructions. A small volume of the exosome sample (e.g., 5–20 µL) was added to the wells of an ELISA plate or test tube. The Bradford reagent (e.g., 200 µL) was then added. The mixture was thoroughly mixed and incubated at room temperature for 5–10 min to allow the reaction to occur. Optical density (OD) measurements were measured at a wavelength of 595 nm using a spectrophotometer. The standard curve was constructed based on the OD values of the BSA standard solutions (OD595 nm versus BSA concentration). The protein concentration in the exosome sample was calculated using the equation derived from the standard curve.

### 3.14. miRNA 203A, miRNA 203B, and miRNA 3196 Quantification and Determination in Exomes

To detect and quantify miRNAs such as miRNA 203A, miRNA 203B, and miRNA 3196 in exosomes, real-time PCR was employed. This is a powerful tool for studying miRNAs in exosomes, with potential applications in disease diagnosis, personalized treatment, and the development of novel therapies. Total RNA was extracted from 100 µL of the sample using the RNDNAAprep-MAGBEAD kit manufactured by Nam Khoa. The extracted solution was recovered in a volume of 100 µL. cDNA Synthesis: 15 µL of the extracted solution was used for cDNA synthesis with the iScript cDNA Synthesis kit from Bio-Rad, utilizing random hexamer primers (6-nucleotide random sequences). The resulting cDNA product contains cDNAs synthesized from miRNAs. Real-Time PCR: 5 µL of cDNA derived from miRNAs was quantified using real-time PCR with a long primer sequence that included a forward complementary sequence for the cDNA, a probe-complementary sequence in the middle, and a reverse complementary sequence for another primer. During real-time PCR, the cDNA product was diluted at four concentrations, (i) undiluted, (ii) 1/10, (iii) 1/100, and (iv) 1/1000, to prevent inhibition at high concentrations due to the presence of non-target miRNA-derived cDNAs. The miRNA quantification of miRNA-derived cDNA was achieved by performing parallel reactions with standard solutions containing DNA analogous to the miRNA cDNA.

### 3.15. Exosomes Flowcytometry CD Markers Identification

A total of 4 µL if exosomes and 1 µL of aldehyde/sulfa latex beads (4% *w*/*v* 4 µm; Invitrogen, Waltham, MA, USA) were added into the 1.5 mL reaction tube (Greiner Bio-One, Frickenhausen, Germany) and incubated for 15 min at room temperature, and 1 mL PBS (Gibco, USA) was added to the whole sample. The mixture was left overnight in ThermoMixer C (Eppendorf, Germany), using the mixing and cooling mode. The following morning, we collected the exosome–bead pellets by centrifugation at 4000× *g* for 10 min, 4 °C, using a cooling 5430R centrifuge (Eppendorf, Germany). We discarded the supernatant carefully and we added 1 mL PBS (Gibco, USA) into the tube; we resuspend the pellets. The tube was moved and centrifuged at 4000× *g* for 10 min at 4 °C. The pellets were washed with PBS, resuspended gently, and separated by using antibodies. We used 2 µL antibodies for each samples. Specific antibodies were conjugated with VioBlue, used for the detection of exosomal CD9, with FITC used for CD63 and APC used for CD81. All antibodies were purchased from Miltenyi Biotec (Rhineland, Germany). The whole solution (exosome–bead and antibodies) was incubated for at least 30 min at 4 °C in a dark cabinet; 500 µL PBS was added and then centrifuged at 4000× *g* for 10 min at 4 °C. We proceeded to discard the supernatant carefully and an additional 500 µL PBS was poured. The samples were analyzed by using MoFlo Astrios EQ, Cell Sorter (Beckman Coulter, USA).

### 3.16. The Exosomes for the In Vivo Experiment on Mice Model Exposed to UVB

This study aimed to evaluate the skin protective efficacy of exosomes in a UV-damaged Swiss Albino mouse model, thereby exploring their potential application in treating skin damage. Healthy male Swiss Albino mice, aged 6–8 weeks and weighing approximately 40 g, were sourced from the Pasteur Institute in Ho Chi Minh City. The concentration of exosomes used in mice was 50 μg/mL. The solvent used for exosome dilution was Xanthan gum 0.1% (Beijing Hongtai Tianshun Chemical Co., Ltd., Beijing, China). The volume of liquid (diluted) was 20 μL/mouse/turn. The mice were acclimatized for three days prior to experimentation. At each time point (2 weeks and 4 weeks), three mice were assigned per group. With three experimental groups evaluated at two points, a total of 18 mice were used, ensuring experiments were repeated at least three times. The outcome assessment was generated using the Agrawal criteria score (Table 1 and Table 2). The exosome suspension, post-isolation, was submitted for skin irritation testing at the VNTEST Center for Testing and Quality Certification, following the ISO 10993-10:2021 standard [67], and no skin irritation observed.

### 3.17. Statistical Analysis

The statistical analysis for the in vitro experiments was executed calculating the mean and standard deviation (SD) for each group both in vitro and in vivo. The overall significant difference between the averages of multiple groups was evaluated using ANOVA. Significant differences between individual means were determined using Tukey’s test. Differences were considered statistically significant at *p* < 0.001 and an F-value of F > 2.5.

## 4. Results

### 4.1. Characterization of Human AT-MSCs and Derived Exosomes

**Figure 1:**
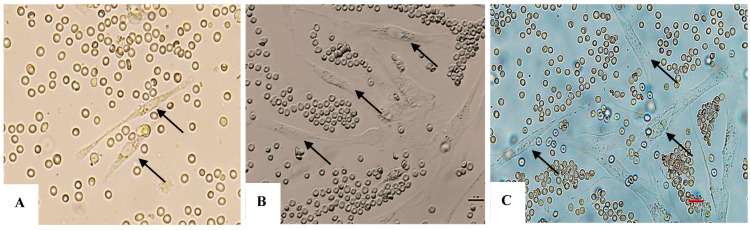
(**A**–**C**): AT-MSCs at day 4, 5, and 6; there are MSCs with typical spindle-like fibroblast shapes (black arrows).

**Figure 2:**
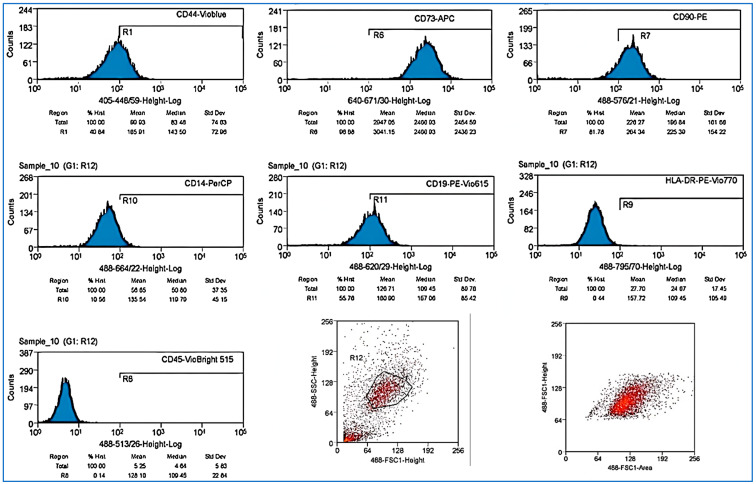
Results of evaluation using flow cytometry to identify AT-MSCs CD-44, CD-73, CD-90, CD-19, and CD14^low^, while showing negative results for CD45 and HLA-DR.

AT-MSCs were recognized by their morphology and through flow cytometry, where MSCs were distinguished by their fibroblast fusiform shape, elliptical nucleus, colony-forming unit, and by their CD markers CD-44, CD-73, CD 90, CD-19, and CD14^low^, while being negative for CD45 and HLA-DR (Figure 1A–C and Figure 2). The characterization of exosomes was detected by their size (100 nm) and by their cup-shaped spheroidal morphology through SEM.

### 4.2. Morphological Assessment of Exosomes Using FE-SEM Electron Microscopy

**Figure 3:**
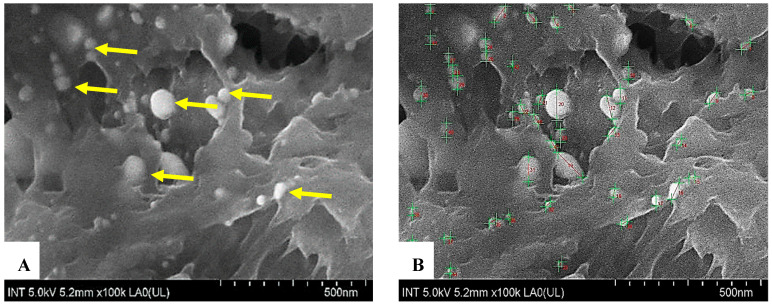
FE-SEM images of exosomes. Using a scanning electron microscope to observe the morphology and size of exosome vesicles revealed that these vesicles are spherical (yellow arrows, (**A**)), with an average size of approximately 40 nm, falling within the 30–200 nm range specified by ISEV standards (**B**).

A field emission scanning electron microscope (FE-SEM) (SU, 8010, Hitachi, Tokyo, Japan) is used to observe the morphology and assess the initial size of exosomes. Exosomes exhibit a spherical shape with sizes ranging from 30 to 200 nm. The size of these vesicles was measured using the NIS-Element AR 5.21.01 64 bit image processing software (Nikon, Tokyo, Japan), yielding an average value of approximately 40 nm, which falls within the ISEV standard range. These observations showed that the exosome suspension product, after completing the isolation process, held macrovesicles with a morphology and size consistent with the criteria for exosome identification (Table 3 and Table 4). (Figure 3A,B).

#### Evaluation of Biological Parameters

The microbial limits were assessed for sterility according to the Vietnam Pharmacopoeia V (DĐVN V). 

**Table 3:** The results met the following criteria.

-Total Aerobic Microbial Count:	≤1000 CFU/g or CFU/mL
-Total Yeast and Mold Count (*)	<10 CFU/g
-*Pseudomonas aeruginosa*:	Absent in 0.1 g (0.1 mL) of the test sample.
-*Staphylococcus aureus*:	Absent in 0.1 g (0.1 mL) of the test sample.
-*Candida albicans:*	Absent in 0.1 g (0.1 mL) of the test sample.

* A value of <10 CFU/g is considered not detected.

### 4.3. Size Evaluation of Exosomes Using Dynamic Light Scattering (DLS)

**Figure 4:**
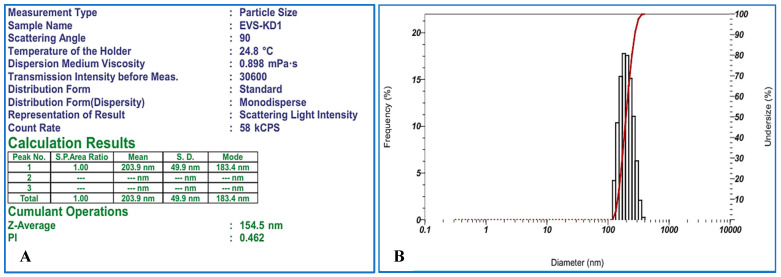
(**A**,**B**) Exosome particle size measurement by Dynamic Light Scattering (DLS). The measurement results indicate that exosome particles have an average size of approximately 154.5 nm, falling within the 30–200 nm range according to ISEV standards. (**B**) The DLS measurements demonstrate that the exosomes have an average size of approximately 154.50 nm, which is within the typical range for exosomes (30–200 nm). This confirms that the sample contains particles with characteristics corresponding to exosomes, without contamination from larger extracellular vesicles such as microvesicles (>200 nm). The red line shows a cumulative distribution curve that matches the ‘Subsize (%)’ axis on the right side of the graph. This curve displays the total percentage of particles with diameters at or below a certain value. At each point on the red curve, the *x*-axis shows the particle diameter, and the right y-axis shows the percentage of the sample with diameters up to that value.

The scattering of a laser beam passing through the exosome-containing suspension revealed a size ranging from 30 to 200 nm (Figure 4A,B).

### 4.4. Assessment of Surface Marker Expression Using Flow Cytometry

**Figure 5:**
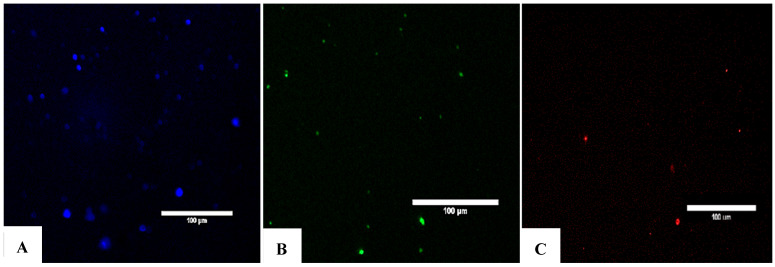
Detection of exosomes as evidenced by the expression of the canonical markers CD9, CD63, and CD81. (**A**) Immunofluorescence staining of CD9. (**B**) Immunofluorescence staining of CD63. (**C**) Immunofluorescence staining of CD81. To evaluate the presence of exosomes in the product suspension, exosomes with fluorescently labeled antibodies were stained against CD9, CD63, and CD81 after incubating the vesicles with beads for flow cytometry preparation. The results were observed under a confocal fluorescence microscope, as shown above. (**A**) Fluorescent staining with anti-CD9 antibody. (**B**) Fluorescent staining with anti-CD63 antibody. (**C**) Fluorescent staining with anti-CD81 antibody. Colors indicate the density of events, such as exosomes, on the flow cytometry plot. The provided percentages—91.36% for CD9, 81.06% for CD63, and 64.73% for CD81—represent the proportion of exosomes within a specific gated region, likely R1, that are positive for each of these markers.

**Figure 6:**
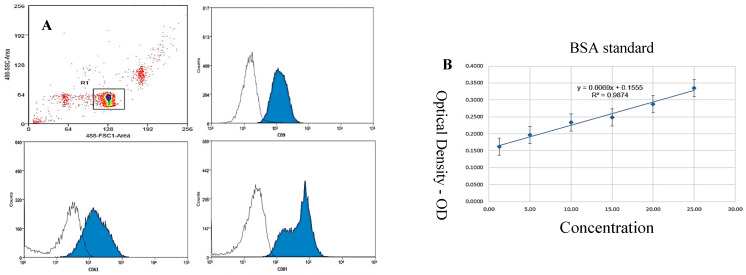
(**A**,**B**): Flow cytometry results for the expression of characteristic CD9, CD63, and CD81 markers for exosome identification. The results indicate the presence of these markers on vesicles within the exosome product suspension, with CD9 expression at 91.36%, CD63 at 81.06%, and CD81 at 64.73%. The observations confirm the expression of these markers in the exosome product suspension post-isolation. To quantify the presence of these characteristic exosome proteins, flow cytometry was employed to assess the expression of CD9, CD63, and CD81 markers. (**B**) Illustration of results from the Bradford assay for quantifying protein concentration in the exosome suspension (x = by absorption; y = concentration). Bradford assay for quantification of protein concentration in the exosome suspension. The calibration curve is depicted with absorbance on the x-axis and protein concentration on the *y*-axis. The results provide a quantitative assessment of protein levels within the exosome preparation. Colors indicate the density of events, such as exosomes, on the flow cytometry plot. The provided percentages—91.36% for CD9, 81.06% for CD63, and 64.73% for CD81—represent the proportion of exosomes within a specific gated region, likely R1, that are positive for each of these markers.

Fluorescently labeled antibodies (Detection Reagent Gibco) are conjugated to exosome surface proteins and analyzed using a flow cytometry system (Beckman Coulter), with a CD panel including the following markers and confirming the expression of the following: exosome–human CD9, exosome–human CD63, and exosome–human CD81 (Figure 5 and Figure 6).

### 4.5. Quantification of Protein Content in Exosome Suspension Using the Bradford Method

**Figure 7:**
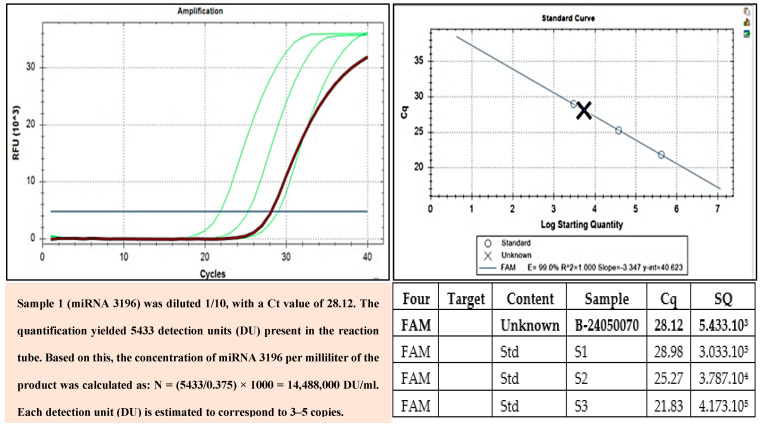
The RT-PCR confirmed the presence of miRNA-3196 in exosome samples. miRNA-3196 is recognized for its role in regulating biological pathways associated with inflammation, metabolism, and cancer. miRNA-3196 is implicated in the regulation of biological pathways related to inflammation, metabolism, and cancer. RT-PCR analysis confirmed the presence of miRNA-3196 in the exosome samples. Green curves: These are the amplification curves of the target miRNA from different replicates. Red or dark curve: This represents the negative control or a baseline/reference curve.

The protein content of the exosome suspension was quantified using the Bradford method, a widely used procedure for protein quantification based on the color change in Coomassie Brilliant Blue G-250 dye upon the binding to proteins (Figure 7).

## 5. Exosome Characterization Outcomes

Exosome products were produced and provided by the Biomedical Research Center of Pham Ngoc Thach University of Medicine of Ho Chi Minh City, Vietnam. The products were transported in cold boxes to the Vietnam Institute of Clinical Microbiology Research and Development (VCM). Here, the products were kept at −80 °C until testing. The exosomes obtained after isolation were evaluated based on morphological characteristics and surface marker expression according to the International Society for Extracellular Vesicles (ISEV) standards.

### 5.1. Exosome Concentration Determined by the Bradford Method

Based on the evaluation of morphology, size, and the expression of characteristic proteins, we can confirm the successful isolation of exosomes from the mesenchymal stem cell culture medium. Subsequently, to assess the concentration of exosomes in the product suspension, the Bradford protein assay was employed to quantify the protein content in the exosome suspension using the equation y = 0.0069x + 0.1555 (R^2^ = 0.9874) (Figure 6B).

### 5.2. Quantification Results of Specific miRNAs Present in Exosomes

**Figure 8:**
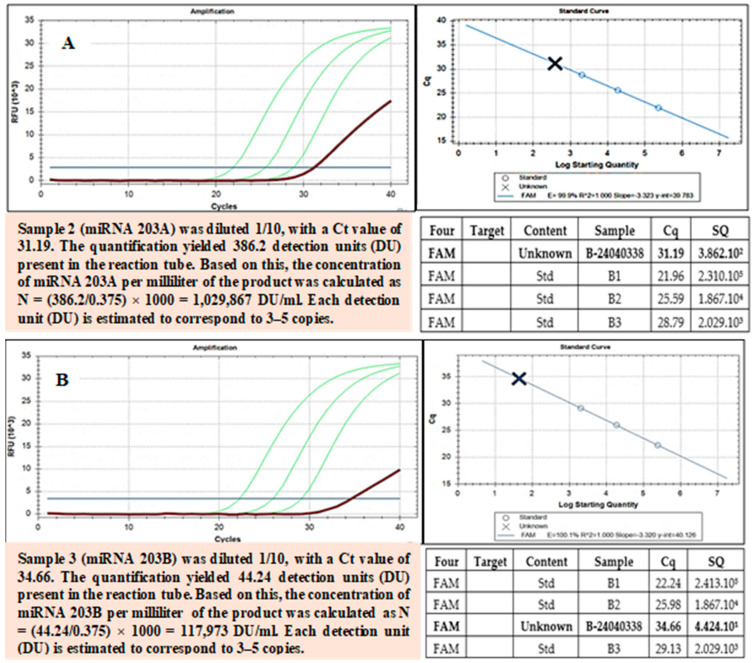
(**A**,**B**) miRNA-203 is a key regulator of cellular differentiation and is implicated in conditions such as cancer, chronic inflammation, and tissue regeneration. RT-PCR confirmed the presence of miRNA-203 (**A**,**B**). Green curves: These are the amplification curves of the target miRNA from different replicates. Red or dark curve: This represents the negative control or a baseline/reference curve.

**Figure 9:**
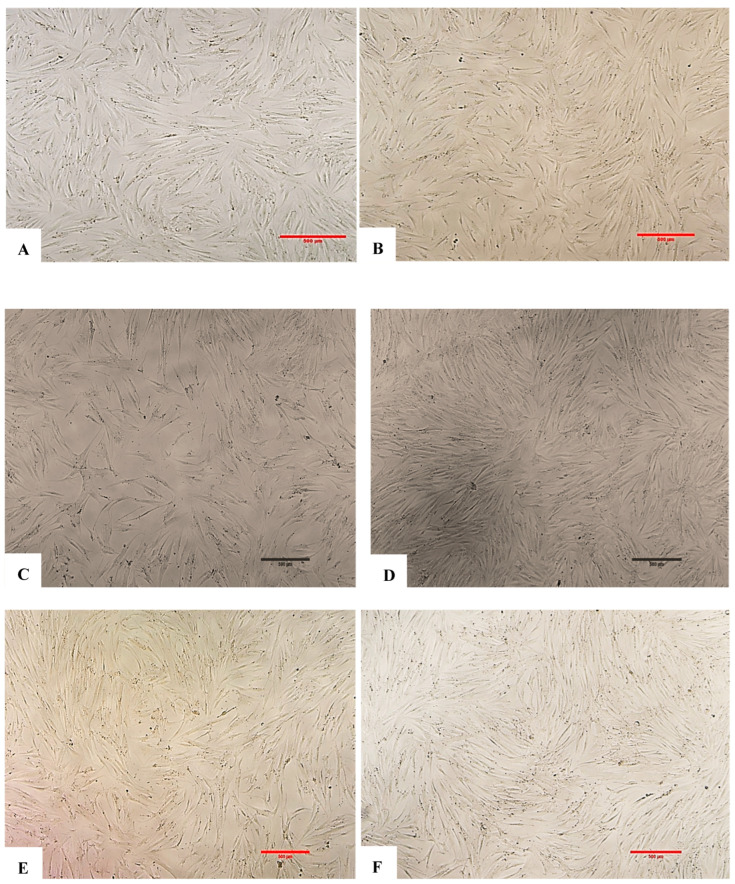

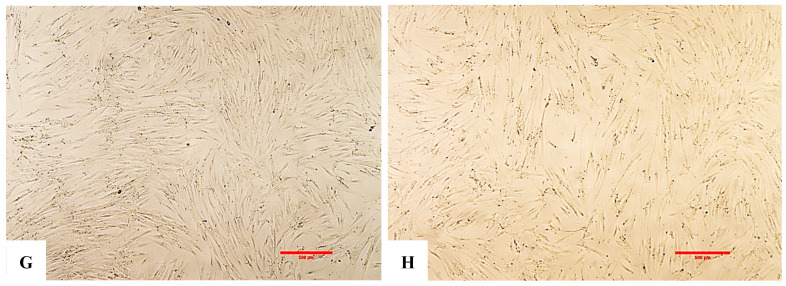
(**A**,**B**) Representative images of fibroblasts captured using an inverted microscope at 4× magnification. (**A**) shows the negative control (no exosomes and no H_2_O_2_), while (**B**) represents the positive control or EXO-0 condition (no exosomes + H_2_O_2_). A marked difference in cell density was observed between the two groups, with the negative control displaying significantly higher cell populations compared to the positive control treated with H_2_O_2_ (*p* < 0.001, F > 2.5). (**C**) (EXO-0.1) corresponds to cells treated with 0.1 µg/mL exosomes + H_2_O_2_, while (**D**) (EXO-0.5) represents cells treated with 0.5 µg/mL exosomes + H_2_O_2_. A clear difference in cell density was observed between them, with (**C**) exhibiting significantly lesser cell populations compared to (**D**) (*p* < 0.001, F > 2.5). (**E**) (EXO-1) corresponds to cells treated with 1 µg/mL exosomes + H_2_O_2_, while (**F**) (EXO-5) represents cells treated with 5 µg/mL exosomes + H_2_O_2_. A pronounced difference in cell density was observed between them, with (**F**) exhibiting significantly higher cell populations (*p* < 0.001, F > 2.5). (**G**) shows the negative control (no exosomes and no H_2_O_2_), while (**H**) represents the positive control or EXO-0 condition (no exosomes + H_2_O_2_). A significant difference in cell density was observed, with the negative control (**G**) displaying markedly higher cell populations compared to the positive control (**H**) (*p* < 0.001, F > 2.5).

**Figure 10:**
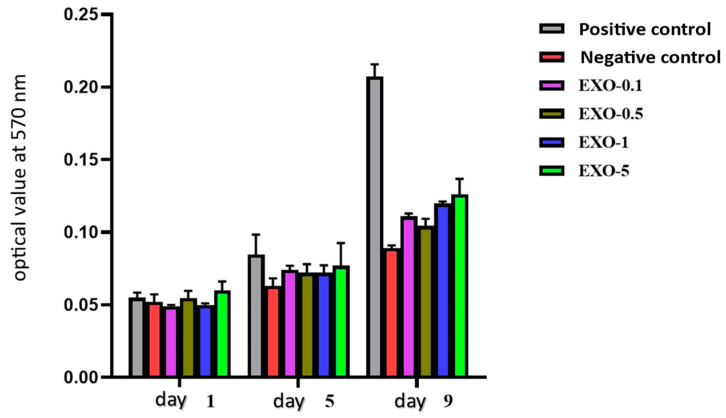
Graph depicting fibroblast proliferation over time across different experimental conditions. Fibroblast proliferation increased following treatment with exosomes at varying concentrations, indicating that exosomes confer protective effects against H_2_O_2_-induced cellular oxidative stress. The data suggested that exosome concentration supports fibroblast proliferation over time, with proliferation increasing progressively from lower (0.1 µg/mL) to higher (5.0 µg/mL) concentrations. Notably, the 5.0 µg/mL concentration yielded the highest proliferation rate among the exosome-treated groups by day 9.

The miRNAs encapsulated within exosomes have been quantified, and we confirmed three types of miRNA in the exosome suspension using RT-PCR: 396, 203A, and 203B (Figure 8, Figure 9 and Figure 10).

### 5.3. Evaluation of Physical and Biological Parameters of Exosome Suspension Post-Isolation

#### 5.3.1. Evaluation of Physical Parameters

Sensory assessment of color and odor: The suspension was transparent, colorless, and odorless. Volume Uniformity: The volume was 1.0 mL of exosome suspension per tube, with no volume less than that indicated on the label. The pH level ranged from 7.0 to 7.4. The testing method complies with the Vietnam Pharmacopoeia V (DĐVN V), Appendix 6, Section 6.2.

#### 5.3.2. Assessment of Heavy Metal Presence

The limits of heavy metals were determined according to the ASEAN ACMTHA05 method, meeting the following standards:

+ Mercury (Hg): Not exceeding 1 mg/kg or 1 mg/L (1 ppm).
+ Lead (Pb): Not exceeding 20 mg/kg or 20 mg/L (20 ppm).
+ Arsenic (As): Not exceeding 5 mg/kg or 5 mg/L (5 ppm).
+ Cadmium (Cd): Not exceeding 5 mg/kg or 5 mg/L (5 ppm).

#### 5.3.3. Assessment of Skin Irritation

The skin irritation potential of the exosome suspension was evaluated according to ISO 10993-10:2021.

## 6. In Vitro Quantification of ROS and Telomeres Before and After Exposure to H_2_O_2_ and SA-Β-Galactosidase Exosomes

**Figure 11:**
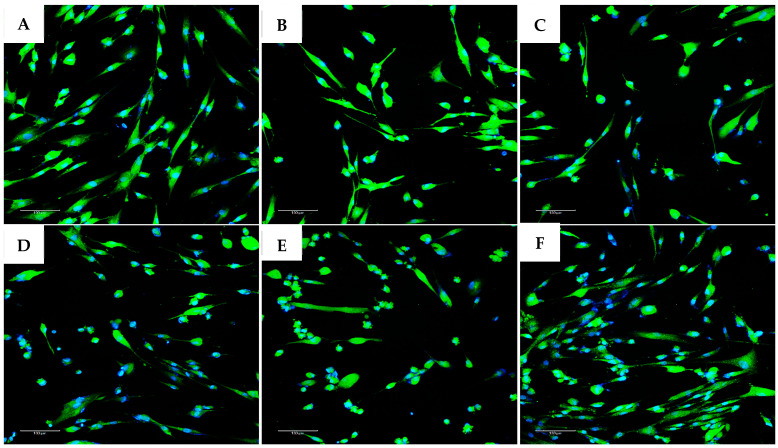
Results of evaluating ROS expression in fibroblasts across different exosome treatment conditions. (**A**) Control group. (**B**) Positive control group. (**C**) EXO-0.1 group. (**D**) EXO-0.5 group. (**E**) EXO-1.0 group. (**F**) EXO-5.0 group. Images were captured at 10× objective magnification. Cells expressing ROS exhibit blue fluorescence. The results indicated that exosomes at a concentration of 5.0 µg/mL (**F**) allowed the lowest levels of ROS expression, suggesting that fibroblasts in this experimental group had the least damage from oxidizing agents, demonstrating the highest protective capacity and lowest oxidative stress among the conditions tested.

**Figure 12:**
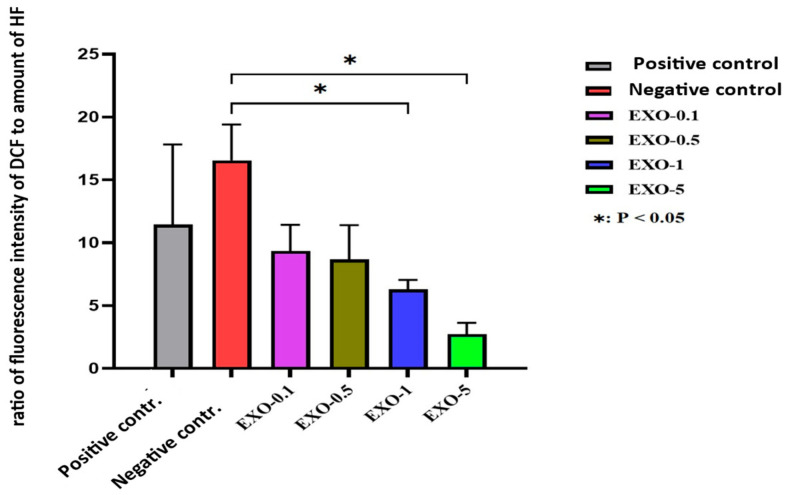
Graph evaluating ROS expression in fibroblasts across different exosome treatment conditions (*p* < 0.05).

**Figure 13:**
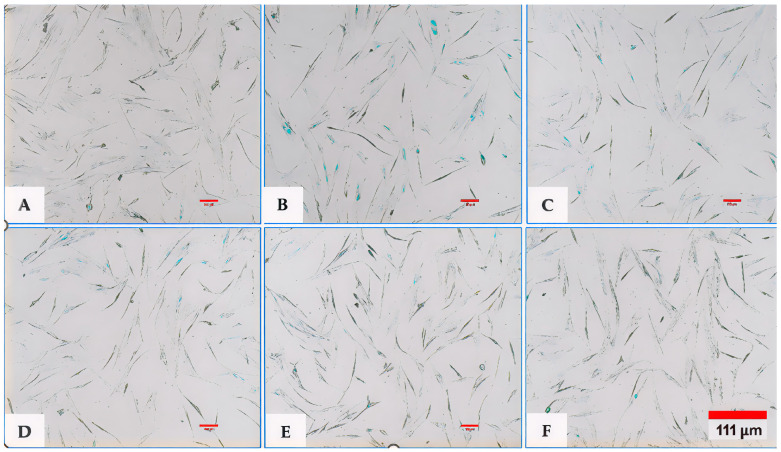
Assessment of fibroblast senescence through SA-β-galactosidase (SA-β-Gal) expression under different experimental conditions. (**A**) Negative control; (**B**) positive control; (**C**) EXO-0.1; (**D**) EXO-0.5; (**E**) EXO-1.0; (**F**) EXO-5.0. Images were captured at 10× magnification. Cells positive for SA-β-Gal expression are identified by blue/green cytoplasmic staining. Cells positive for SA-β-Gal expression exhibit blue/green staining in the cytoplasm.

**Figure 14:**
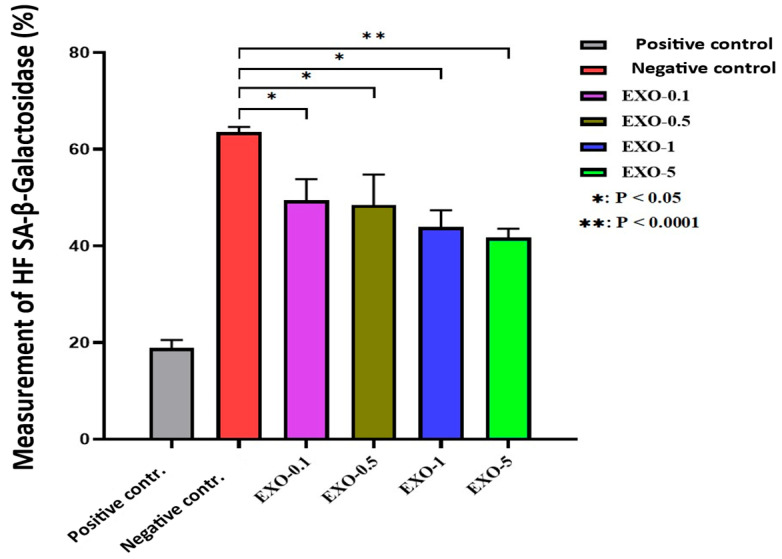
Graph illustrating SA-β-galactosidase expression in fibroblasts under different exosome treatment conditions. Statistically significant differences are indicated (* *p* < 0.05, ** *p* < 0.0001).

**Figure 15:**
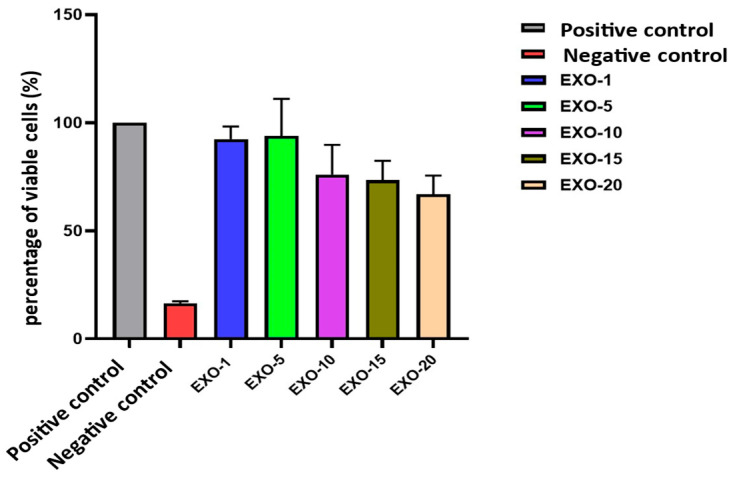
Graph illustrating viability assessment on fibroblasts across different exosomes’ gradient concentrations. Based on these findings, exosomes at a concentration of 5 µg/mL proved an optimal value compared to the other tested concentrations. At this concentration, cell viability reaches 93.90% (compared to 100% in the control sample).

The use of exosomes in vitro was conducted on fibroblast populations, and the results were evaluated in terms of ROS and telomere length before, during, and after the addition of H_2_O_2_ and SA-β-Galactosidase to induce senescence. Data was statistically significant. Exosomes proved their significant potential in reducing fibroblast senescence by decreasing ROS, with SA-β-Galactosidase expression favoring telomere length and stability, therefore improving cell survival (ANOVA test showed a *p*-value < 0.001, which indicates a significant difference between the means of the groups; the Tukey test confirmed these significant differences between many of the pairs of groups, with *p*-value > 2.5) (Figure 11, Figure 12, Figure 13, Figure 14 and Figure 15).

### 6.1. Fibroblast Morphological Assessment with Exosomes and H_2_O_2_

Except for the negative control group, the remaining experimental groups were stressed by H_2_O_2_ and exhibited morphological changes, tending to swell more than the control group (no H_2_O_2_ treatment). Each image was named, with experimental groups as follows: control (negative control); EXO-0 (positive control); EXO-0.1 (0.1 µg/mL exosomes + H_2_O_2_); EXO-0.5 (0.5 µg/mL exosomes + H_2_O_2_); EXO-1 (1 µg/mL exosomes + H_2_O_2_); EXO-5 (5 µg/mL + H_2_O_2_); negative control (no exosomes + no H_2_O_2_); positive control or EXO-0 (no exosomes + H_2_O_2_). Cells expressing ROS exhibit blue fluorescence. The results indicated that exosomes at a concentration of 5.0 µg/mL (Figure 13F) resulted in the lowest levels of ROS expression, suggesting that fibroblasts in this experimental group experienced the least damage from oxidant agents, demonstrating the highest protective capacity and the lowest oxidative stress among the conditions tested (Figure 11A–F and Figure 12) (Table 4).

**Table 4:** Results of evaluating ROS expression in fibroblasts following oxidative stress by H_2_O_2_ with exosome treatment.

No.	Experimental Group	Fluorescence Intensity Ratio Per Fibroblast	Mean ± SD
1	Negative Control	11.44	6.39
2	Positive Control	16.54	2.87
3	Exo-0.1	9.35	2.08
4	Exo-0.5	8.66	2.74
5	Exo-1.0	6.28	0.76
6	Exo-5.0	2.73	0.91

The results from the table are graphically represented below:

### 6.2. Results of Fibroblast Senescence Through SA-β-Galactosidase Expression (Senescence-Associated Beta-Galactosidase)

The SA-β-gal (SA-β-Gal) serves as a hallmark indicator of cellular senescence. It is strongly expressed at pH 6.0 in senescent cells, with its activity increasing in response to stressors such as oxidative stress, DNA damage, or prolonged cell division (replicative senescence). The percentage of fibroblasts expressing SA-β-Gal varied significantly depending on exposure to exosomes: the higher the amount used, the lower the expression of SA-β-Gal by fibroblasts (Figure 13A–F and Figure 14) (Table 5).

**Table 5:** The results of evaluating fibroblast senescence are presented.

No.	Experimental Group	Percentage of Fibroblasts Expressing SA-β-Galactosidase (%)	Mean ± SD
1	Negative Control	18.90	1.66
2	Positive Control	63.53	1.11
3	Exo-0.1	49.43	4.43
4	Exo-0.5	48.48	6.29
5	Exo-1.0	43.92	3.48
6	Exo-5.0	41.74	1.8434

The results from the table are graphically represented below:

### 6.3. Results of Viability Assessment of Exosomes on Fibroblasts

**Figure 16:**
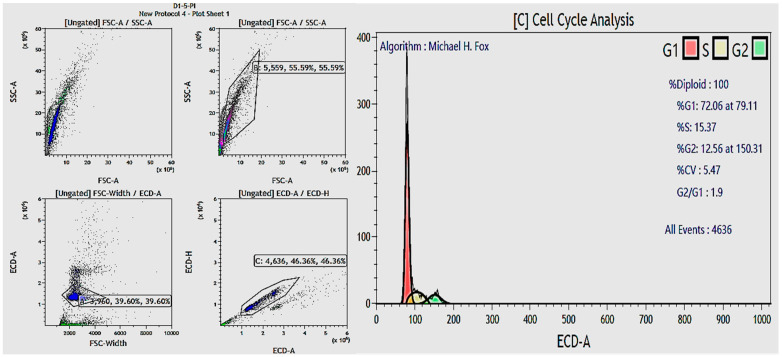
Gating and cell cycle analysis results of the fibroblast population in the exosome 5.0 µg/mL treatment group.

Exosomes promoted fibroblast viability and migration. Cell viability was evaluated in fibroblasts with different exosome concentration gradients. Based on these results, exosomes at a concentration of 5 µg/mL showed the best value compared to the other concentrations tested. At this concentration, cell viability reached 93.90% (compared to 100% in the control sample) (Figure 14, Figure 15 and Figure 16) (Table 6).

### 6.4. Results of Fibroblast Cell Cycle Evaluation

**Figure 17:**
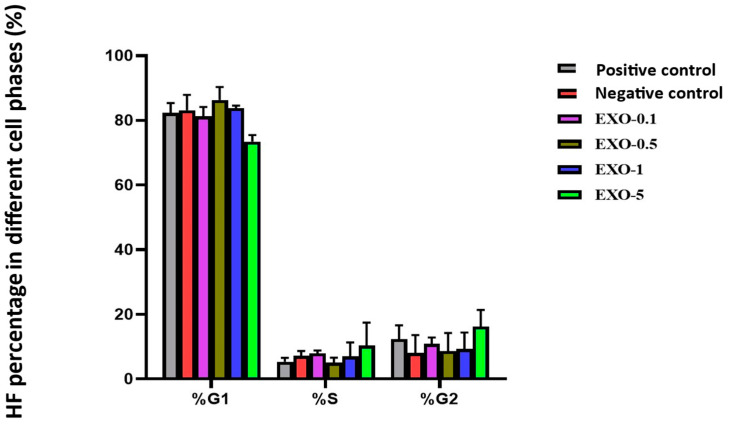
Graph evaluating the percentage distribution of fibroblasts in G1, S, and G2 phases across different exosome treatment conditions. G0/G1 phase: Cells in this phase are considered healthy and at rest, with round nuclei. S phase: This phase is characterized by DNA replication, and cells in this phase may have elongated nuclei. G2/M phase: This phase involves preparation for cell division and mitosis. Cells in this phase may have dividing nuclei. The EXO-5 showed the best performance in both S and G2 phases by promoting entry into the S and G2 phases of the cell cycle through transfer of miRNAs 203 and 3196 to recipient cells. By resolving cell cycle arrest in the G1/S phase, these miRNAs ease cell cycle progression and entry into S and G2 phases, leading to proliferation.

Under the tested experimental conditions, treatment with 5.0 µg/mL exosomes yielded the highest proportions of fibroblasts in the S phase (10.32%) and G2 phase (16.21%). Cell cycle analysis during the exosome treatment has shown their ability to stimulate cell proliferation, particularly in the S and G2 phases of the fibroblast interphase, which are related to DNA replication and the preparation for cell division and mitosis (Figure 17) (Table 7). This underscores the potential of exosomes in regenerative medicine and anti-aging therapies, paving the way for future research and clinical development.

The research results are graphically represented below:

### 6.5. Telomere Length in Fibroblasts After Exposure to Oxidative Stress with Exosome Treatment

**Figure 18:**
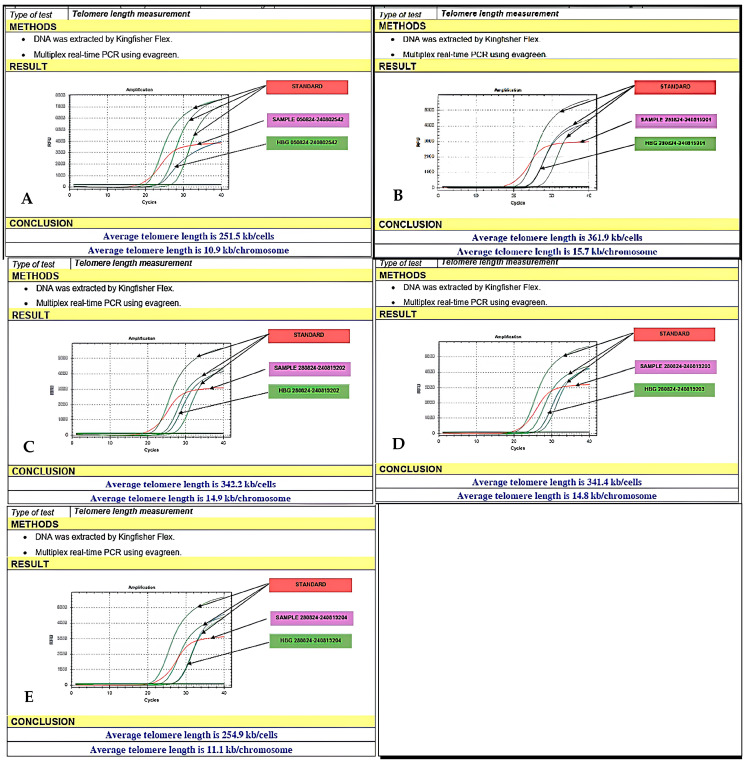
(**A**,**B**) Fibroblasts without any exposure; this served as a control to assess the baseline effect of exosomes on fibroblast cells in the absence of oxidative stress; telomere length was 10.9 kb. Fibroblasts were exposed to exosomes for 24 h, followed by 200 µM H_2_O_2_ treatment for 90 min; telomere length was 15.7 kb. (**C**,**D**) Fibroblasts were exposed to treatment with 200 µM H_2_O_2_ for 90 min; subsequently, exosomes were added for 24 h; the telomere measured 14.9 kb. Figure 11: Fibroblasts were exposed to exosomes only for 24 h; telomere measured 14.8 kb. (**E**) Fibroblasts were exposed to 200 µM H_2_O_2_ for 90 min; the resulting telomere length was 11.1 kb.

**Figure 19:**
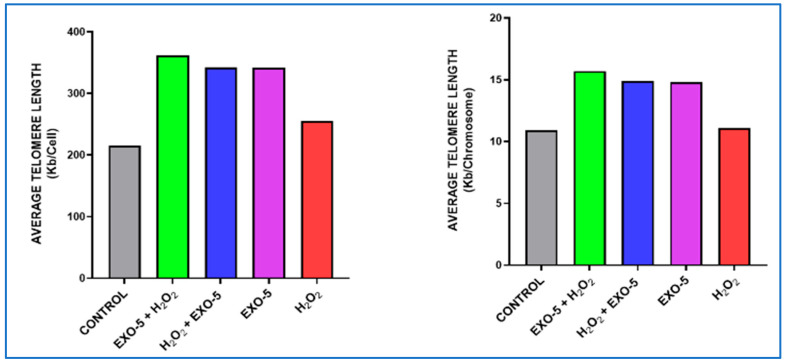
Graph evaluating telomere length per chromosome and per cell across the five experimental conditions. Condition 1: Fibroblast control group. Condition 2: hFs were incubated with exosomes at a concentration of 5.0 µg/mL and subsequently exposed to H_2_O_2_ (200 nM). Condition 3: hFs were incubated with H_2_O_2_ (200 nM) and then were exposed to exosomes at a concentration of 5.0 µg/mL. Condition 4: hFs were exposed to exosomes only at a concentration of 5.0 µg/mL. Condition 5: hFs were exposed to H_2_O_2_ (200 nM). Exosomes generally exert a clear protective and enhancement activity toward cells exposed to H_2_O_2_; eventually, the best gradient was 5.0 µg/mL (green) (*p*-value < 0.001).

**Figure 20:**
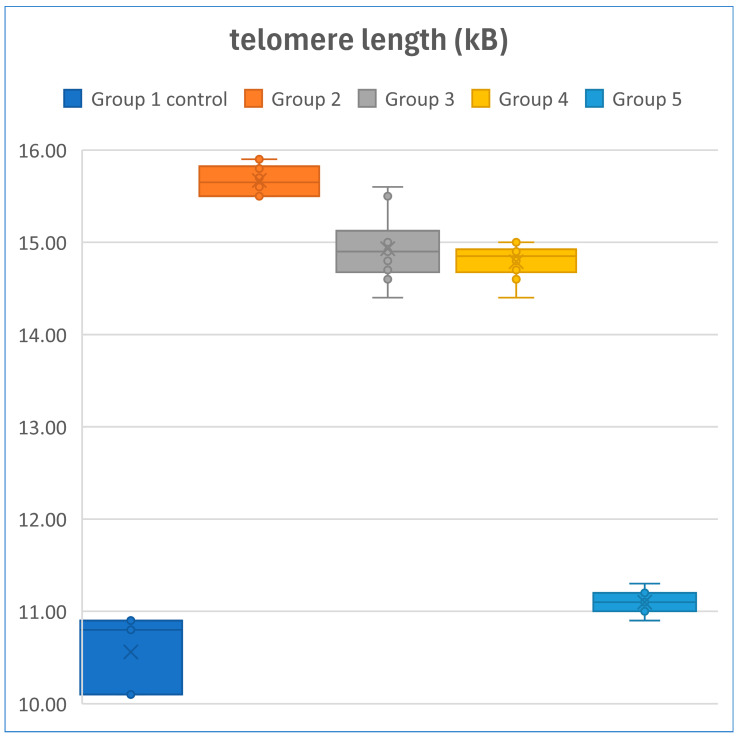
Exosomes caused an increase in telomere content, observed primarily under the influence of the highest concentration tested on each cell sample: control group 1 (dark blue), group 2 (orange), group 3 (gray), group 4 (dark yellow), and exosome dose group 5 (light blue) included in the experiment. Exosomes had a stronger effect on the tested parameter both before and after H_2_O_2_, even at the lowest concentration (*p* value < 0.001).

**Figure 21:**
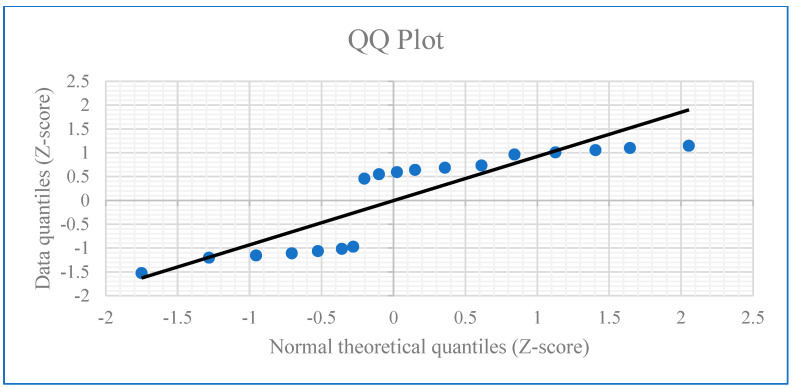
The Q-Q plots indicate that the full sample did not deviate from linearity due to the influence of one sample (Experiment 5); data are normally distributed (*p*-value is < 0.001).

Five experimental setups were used. **Experiment 1 (Control):** fibroblasts were not exposed to exosomes and were not treated with H_2_O_2_, resulting in a telomere length of 10.63 kb. **Experiment 2:** fibroblasts were exposed to exosomes (5 µg/mL) for 24 h, followed by treatment with H_2_O_2_ (200 µM) for 90 min; the telomere length was 15.7 kb. **Experiment 3:** fibroblasts were treated with H_2_O_2_ (200 µM) for 90 min, followed by exposure to exosomes (5 µg/mL) for 24 h; the telomere length was 14.9 kb. **Experiment 4:** fibroblasts were exposed to exosomes only for 24 h, resulting in a telomere length of 14.8 kb. **Experiment 5:** fibroblasts were treated with H_2_O_2_ (200 µM) for 90 min, resulting in a telomere length of 11.1 kb (Figure 18A–E). Significant differences were observed between Group 1 and Groups 2, 3, and 4; between Group 2 and Groups 1, 3, 4, and 5; and between Group 5 and Groups 2, 3, and 4. All group comparisons showed statistically significant differences (Figure 19, Figure 20 and Figure 21).

## 7. The Outcomes of In Vivo Exosome Therapy Performed on Albino Models

### 7.1. Assessment of Skin Healing Rate, In Vivo Procedure, Evaluation of Exosomes Protection Efficacy in Albino Mouse Model and Experimental Design

The assessment of the skin healing rate was based on the comparison of external skin characteristics and internal morphological and structural assessments (histology) of the tested area using the Hagrawal criteria (Table 8). The study results proved that exosomes exert a significant protective and restorative effect on UV-damaged skin in a Swiss Albino mouse model. Notable improvements were seen through external morphological assessments of the test area and histological analyses, underscoring the potential of exosomes as a therapeutic agent for skin damage. Specifically, exosome treatment at a concentration of 50 µg/mL effectively restored skin regions impaired by UVB radiation. UVB radiation adversely impacts the skin by inducing cellular damage, reducing epidermal thickness, and accelerating aging processes. Exposed skin exhibits signs of inflammation, erythema, scaling, and potentially hyperpigmentation or wrinkle formation.

### 7.2. At 2 Weeks

**Figure 22:**
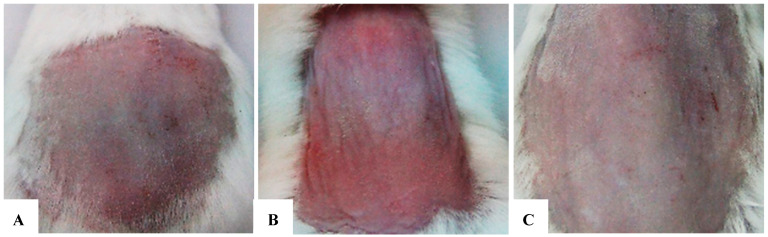
(**A**–**C**) Comparison of morphological assessments at 2 weeks. (**A**) Negative control group. (**B**) Positive control group. (**C**) Research group (treated with exosomes prior to each UVB exposure). After 2 weeks, distinct differences in the test area’s morphology were clear across the three groups, particularly in terms of color, elasticity, and inflammation reduction. The exosome-treated group exhibited substantial improvement compared to the untreated group, confirmed by reduced inflammatory patterns, scaling, and enhanced elasticity. The untreated positive control group showed persistent severe damage, with dry, rough skin and slow recovery. The negative control group kept healthy skin with no signs of damage or inflammation.

The application of exosomes at a concentration of 50 µg/mL yielded a significant efficacy in reducing UVB-induced skin damage. Outcomes at 2 weeks were evaluated based on external morphological criteria of the test area, including skin color, scaling, dryness, elasticity, and firmness. These results are documented in Table 9 and Table 10 (Figure 22A–C).

### 7.3. At 4 Weeks

At 4 weeks into the experiment, the morphological differences between the three groups became even more marked. At week 4, the exosome-treated group showed an almost complete recovery, with smooth, firm skin and minimal signs of damage. The untreated positive control group exhibited lingering damage, with some hyperpigmentation and aging signs, indicating a slower natural recovery without exosomes. The negative control group kept healthy skin with no notable changes (Figure 22A–C) (Table 8).

**Table 8:** Comparison of external morphological assessments; classification according to Agrawal criteria—group 1 (negative control group).

Time Point	Figure	Wrinkle Assessment	Agrawal Grade	Explanation
Week 2	Figure 22A	Smooth skin, no visible lines or creases	Grade 0	No signs of wrinkles; the skin appears youthful and unlined at rest and with movement.
Week 4	Figure 23A	Smooth skin, no visible lines or creases	Grade 0	Skin stays clear and smooth, showing no wrinkle formation even under expression.
Week 6	Figure 24A	Very fine lines appear, only with movement	Grade 0	Wrinkles are minimal and dynamic, seen only during facial movement and not visible at rest.

**Figure 23:**
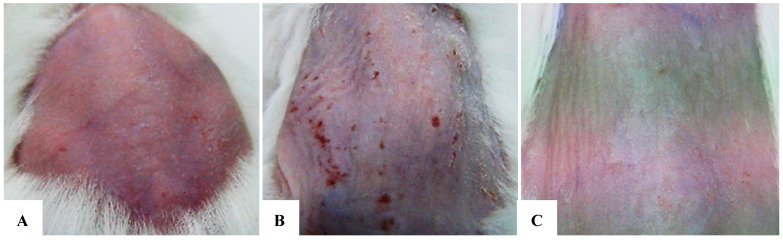
(**A**–**C**) Comparison of morphological assessments at 4 weeks. (**A**) Negative control group. (**B**) Positive control group. (**C**) Research group (treated with exosomes prior to each UVB exposure). The morphological distinctions after 4 weeks were further analyzed and are detailed in Table 9.

**Table 9:** Classification according to Agrawal criteria—group 2 (positive control group).

Time Point	Figure	Wrinkle Assessment	Agrawal Grade	Explanation
Week 2	Figure 22B	Visible lines with mild to moderate depth at rest	Grade 2	Wrinkles are noticeable even without facial movement, forming shallow creases.
Week 4	Figure 23B	Well-defined wrinkles are present, with greater depth and length	Grade 3	Wrinkles are clearly visible and moderately deep, indicating progressing skin aging.
Week 6	Figure 24B	Deep and static wrinkles, with visible furrows and fixed lines	Grade 4	Severe skin aging with permanent, deep wrinkle lines observed clearly at rest.

### 7.4. At 6 Weeks

**Figure 24:**
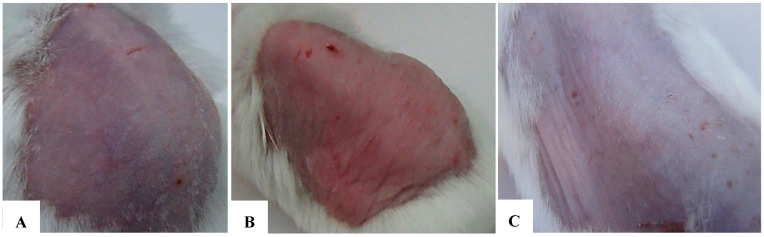
(**A**–**C**) Comparison of morphological assessments at 6 weeks. (**A**) Negative control group. (**B**) Positive control group. (**C**) Research group (treated with exosomes prior to each UVB exposure). The morphological distinctions after 6 weeks were deeply analyzed and are detailed in Table 10.

At 6 weeks into the experiment, the morphological differences between the three groups became even more marked. At week 4, the exosome-treated group showed an almost complete recovery, with smooth, firm skin and minimal signs of damage. The untreated positive control group exhibited lingering damage, with some hyperpigmentation and aging signs, indicating a slower natural recovery without exosomes. The negative control group retained healthy skin with no notable changes (Figure 24A–C) (Table 10).

**Table 10:** Classification according to Agrawal criteria—group 3 (treatment group applied exosome solution prior to each UVB exposure).

Time Point	Figure	Wrinkle Assessment	Agrawal Grade	Explanation
Week 2	Figure 22C	Very fine lines are barely visible and only during facial movement	Grade 1	The skin appears smooth at rest; wrinkles are minimal and dynamic, indicating very early-stage aging.
Week 4	Figure 23C	Mild but visible wrinkle lines are present even at rest	Grade 2	Shallow wrinkles are becoming more defined, visible without expression—early sign of fixed skin changes.
Week 6	Figure 24C	Similar to week 4; wrinkle lines remain mild and have not progressed	Grade 2	No further deepening of wrinkles; exosome treatment may have stabilized skin aging at early stage.

## 8. Assessment of Skin Healing Rate Based on Histological Structure

To assess the efficacy of exosomes, a histological analysis was performed to evaluate structural and functional changes in collagen and elastin between the three experimental mouse groups.

### 8.1. Assessment of Collagen Fiber Structure at 2 Weeks

**Figure 25:**
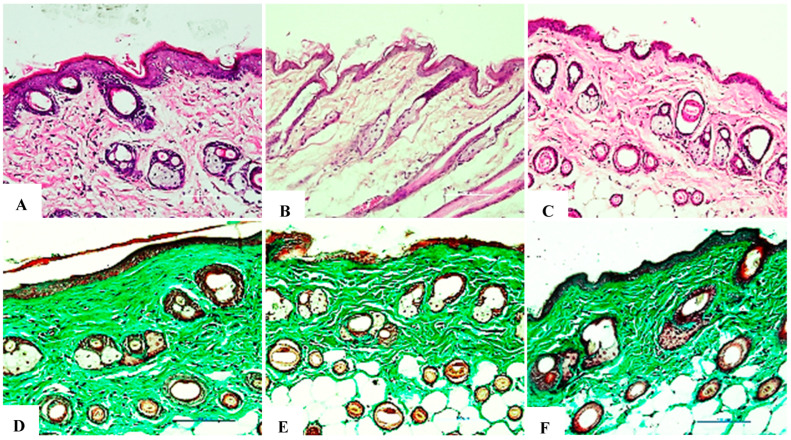
(**A**–**F**). Comparison of histological results for collagen at 2 weeks. (**A**,**D**) Negative control group stained with H&E and Trichrome, respectively (20× objective). (**B**,**E**) Positive control group stained with H&E and Trichrome, respectively (20× objective). (**C**,**F**) Research group (treated with exosomes before each UVB exposure) stained with H&E and Trichrome, respectively (20× objective). The negative control group showed an intact collagen structure reflecting healthy skin; the positive control group showed that collagen density was significantly reduced, with marked structural breakdown due to UVB exposure, which caused inflammation and early signs of aging. The research team demonstrated that collagen density was higher than that of the positive control group, indicating early recovery. Collagenous fibers showed a phase of reorganization but did not reach optimal levels. This suggests that exosomes stimulated collagen regeneration after 2 weeks, although further time is needed for full recovery.

Collagen plays a vital role in maintaining the integrity of the skin’s structure and elasticity. Exposure to UVB rays tends to compromise the complexity of collagen, resulting in loose, wrinkled skin with a reduced ability to regenerate. At week 4, differences in collagen organization became more pronounced between groups. The negative control group showed a well-organized collagen structure, indicative of healthy skin. The positive control group showed a steady decline in collagen with a significant reduction in the structural density of the dermis. The structure was severely damaged, with a disordered and fragmented arrangement. The skin showed clear signs of aging from the 2-week time point, including fibrotic areas and small scars, indicating that prolonged UVB-induced damage cannot repair itself and worsens over time (Figure 25A–F) (Table 11).

### 8.2. Assessment of Collagen Fiber Structure at Week 4

**Figure 26:**
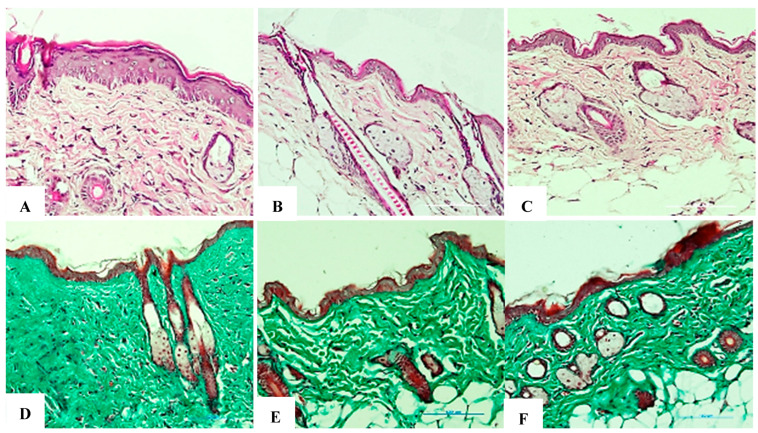
(**A**–**F**). Comparison of histological collagen structure assessments at the 4-week time point. (**A**,**D**) Negative control group stained with H&E and Trichrome, respectively (20× objective). (**B**,**E**) Positive control group stained with H&E and Trichrome, respectively (20× objective). (**C**,**F**) Research group (treated with exosomes prior to each UVB exposure) stained with H&E and Trichrome, respectively (20× objective).

The research group showed an almost complete recovery of collagen density after 4 weeks, with only about a 10% reduction compared to the negative control group. The fiber arrangement improved significantly, with longer and less disorganized fibers. Dermal thickness increased substantially compared to the positive control group, approaching normal levels. No scars or fibrotic tissue were detected, demonstrating the ability of exosomes to promote collagen synthesis and restore skin architecture. The evaluation results are presented in Figure 26A–F and Table 12.

### 8.3. Assessment of Elastin Fiber Structure at Week 2

**Figure 27:**
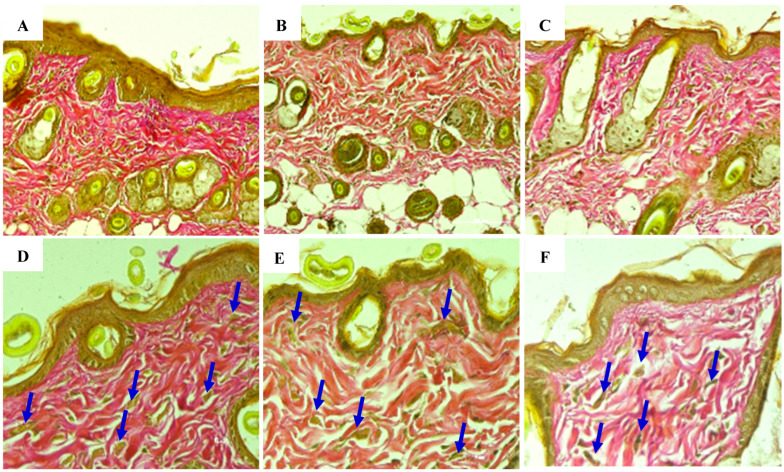
(**A**–**F**) Comparison of elastin histological structure assessments at 2 weeks. (**A**,**D**) Negative control group stained for elastin at 20× and 40× objectives, respectively. (**B**,**E**) Positive control group stained for elastin at 20× and 40× objectives, respectively. (**C**,**F**) Research group (treated with exosomes prior to each UVB exposure) stained for elastin at 20× and 40× objectives, respectively. Elastin fiber structure is indicated by blue arrowheads.

Elastin is a protein essential for maintaining skin elasticity and firmness. When damaged by UVB radiation, elastin begins to deteriorate, and the skin commences losing structural integrity, resulting in sagging and wrinkling. After 2 weeks, elastin recovery remained incomplete; however, initial differences were observed between the three experimental groups. The histological evaluation of elastin structure at 2 weeks is presented in the following figure below (Figure 27A–F) (Table 13).

### 8.4. Anesthesia/Euthanasia

The mice were moved to separate cages and immediately euthanized with 100% CO_2_-displaced air (30% of chamber volume/min) for 5 min; their tissue was then harvested for histological examination.

### 8.5. Assessment of Elastin Fiber Structure at Week 4

Exosomes at a concentration of 50 µg/mL exhibited a positive effect on skin recovery following UVB-induced damage, accelerating the amelioration of inflammation, dryness, and elasticity loss in mice. After 4 weeks of exosome administration, elastin showed clear differences in the three groups, marking a milestone in evaluating the efficacy of the exosome in regenerating elastin, improving skin elasticity, and reducing the damage caused by UVB rays. The negative control group showed a high elastin density with dense and well-organized arranged layers. We observed a well-preserved grade of elasticity with firm skin and no aging signs. No structural damage or alterations in elastin were observed.

**Figure 28:**
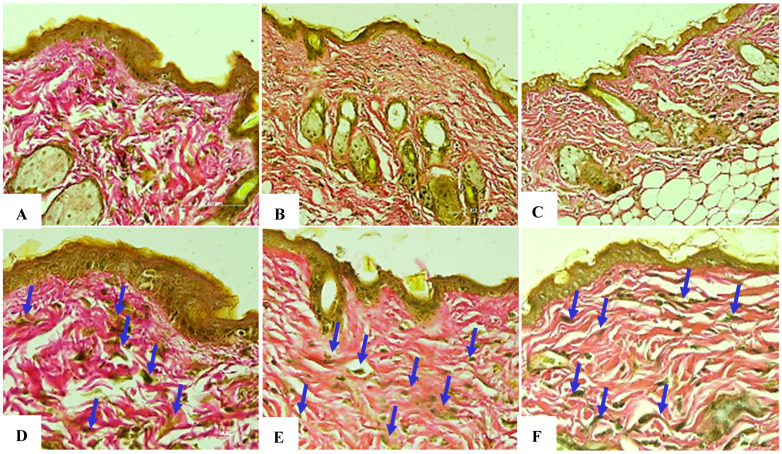

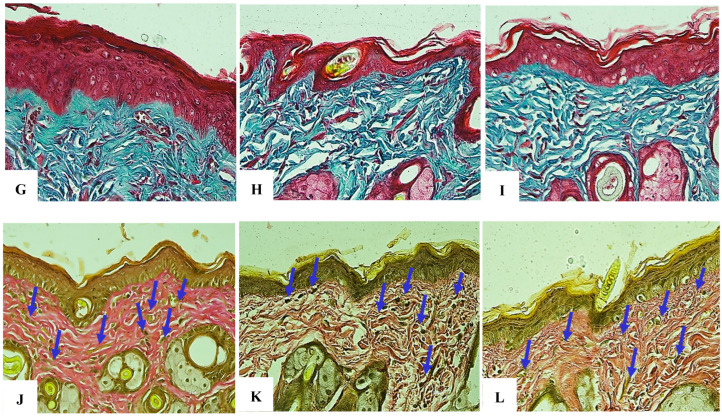
(**A**–**F**) Comparison of elastin histological structure assessments at the 4-week time point. (**A**,**D**) Negative control group stained for elastin at 20× and 40× objectives, respectively. (**B**,**E**) Positive control group stained for elastin at 20× and 40× objectives, respectively. (**C**,**F**) Research group (treated with exosomes prior to each UVB exposure) stained for elastin at 20× and 40× objectives, respectively. Elastin fiber structure is indicated by blue arrowheads. (**G**–**I**) Comparative histological assessment of collagen structure at the 6-week time point. (**G**) Negative control group stained with H&E and Trichrome (40× objective). (**H**) Positive control group stained with H&E and Trichrome (40× objective). (**I**) Exosome-treated group (administered prior to each UVB exposure) stained with H&E and Trichrome, respectively (40× objective). (**J**–**L**) Comparative histological assessment of elastin structure at the 6-week time point. (**J**) Negative control group stained for elastin at 40× magnification. (**K**) Positive control group stained for elastin at 40× magnification. (**L**) Exosome-treated group (administered prior to each UVB exposure) stained for elastin at 40× magnification. Elastin fibers are indicated by blue arrowheads.

**Figure 29:**
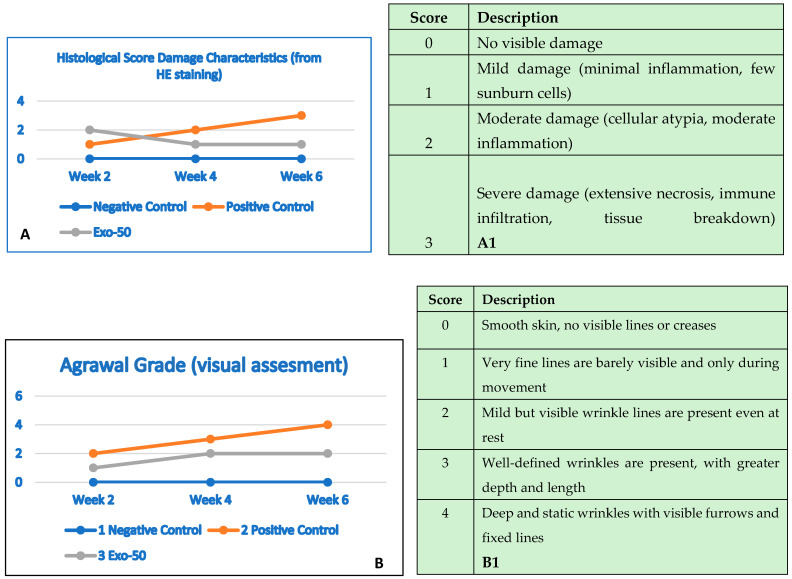

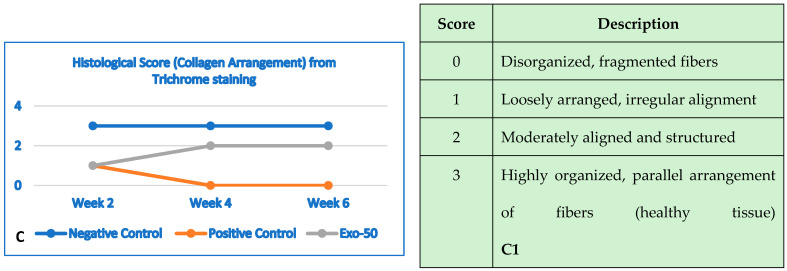
(**A**,**A1**) The negative control group showed no changes in damage characteristics, meaning no damage occurred. In the positive control group, mild damage occurred in the second week, but over time the damage became more severe, resulting in extensive tissue damage by the sixth week. In the “EXO-50” group, moderate damage occurred in the second week. Over time, the damage improved, from moderate to mild by the fourth week, maintaining the status quo. This resulted in an improvement from the second to the sixth week. (**A1**) Histological evaluation based on the histologic wound-healing scoring system. (**B**,**B1**) Negative control group: no visible changes in the skin from week 2 to week 6. Positive control group: at week 2, there were mild but visible wrinkle lines seen even at rest. At week 4, wrinkles were well defined, with greater depth and length. At week 6, skin resulted in deep and static wrinkles with visible furrows and fixed lines. The “EXO-50” group: at week 2, models showed mild wrinkles; improvements were seen at week 4; visible at rest, fine lines were visible only during movements. At week 6, the skin improvements were persistent. (**B1**) Visual evaluation based on the Agrawal criteria, with a gradient ranging from 0 to 4. (**C**,**C1**). The negative control group showed no changes or disruptions in collagen arrangement. In the positive control group, we observed (from week 0 to week 2) a deep deterioration of the collagen fiber arrangement. Further worsening effects were seen at week 2 onward, resulting in disorganization and fragmentation of skin structure fibers. The “EXO-50” group showed significant improvements of the collagen fibers from week 2 onward. At week 4, loosely arranged and irregularly aligned collagen become moderately aligned and structured. Over time, the condition remined stable. (**C1**) Histological evaluation based on the histologic wound-healing scoring system for collagen arrangement evaluated using trichrome staining (score from 0 to 3).

**Figure 30:**
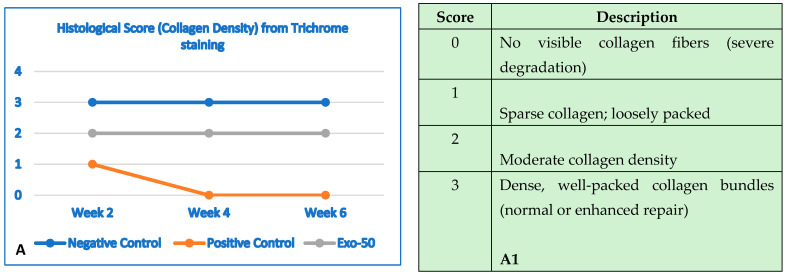

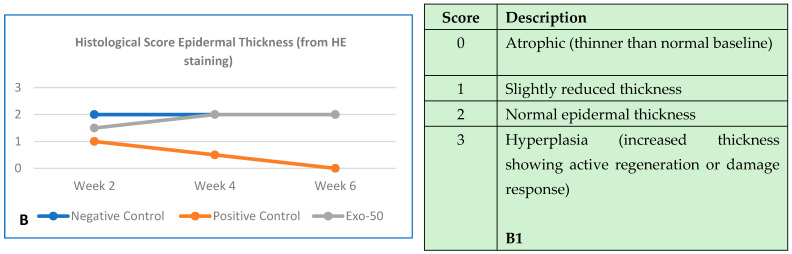
(**A**,**A1**) The negative control group (blue line) showed no changes in collagen structure and organization up to 6 weeks. The positive control group (orange line) showed a gradual and significant reduction in collagen density by the end of 6 weeks. The “exo-50” group showed moderate collagen density at week 2, despite the damage, but did not experience deterioration like the positive control group (gray line). From week 2 onward, density remained moderate with no further degradation. (**B**) Histological evaluation based on the histologic wound-healing scoring system; here, it highlighted collagen density, evaluated by using trichrome staining (score from 0 to 3). (**B**,**B1**) The negative control group (blue line) histology outcomes showed no changes in epidermal thickness. The positive control group (orange line) showed a reduction in thickness starting by the second week throughout week 6. In the “exo-50” group, skin thickness improved in week 4, reaching normal thickness until the end of week 6. (**B**) Histological evaluation based the histologic wound-healing scoring system, based on HE staining (score from 0 to 3).

The positive control group revealed a slight increase in elastin density from the second week, but remained significantly lower than the negative control, indicating a slow self-repair. Overall, the structure remained fragmented and disorganized, with abundant abnormal clusters, demonstrating improper restructuring. Poor elasticity persisted, with sagging skin and reduced firmness, showing a minimal improvement from the second week. Wrinkles became more pronounced, without any reduction, highlighting that prolonged UVB damage cannot fully self-repair within 4 weeks without intervention. The research group showed a substantial recovery of the elastin structure, far superior to the positive control. The fibers were longer, less damaged, and better organized. Skin elasticity improved significantly, approaching normal levels with increased firmness. Wrinkles were significantly reduced, with skin close to its original state and without sagging compared to the positive control. At 4 and 6 weeks, exosomes significantly supported elastin recovery, restoring skin elasticity and substantially mitigating UVB-induced damage (Figure 28A–L, Figure 29A,A1,B,B1,C,C1 and Figure 30A,A1,B,B1) (Table 14 and Table 15).

## 9. Discussion

### 9.1. The Role of Mesenchymal Stem Cells and Derived Exosomes in Anti-Aging Procedures

Aging, in addition to mental and cognitive decline, involves a progressive deterioration of connective tissue, characterized by organ dysfunction and degenerative diseases [68]. These can manifest in early and middle age with a variable progression of clinical signs. The most important of these is inflammation [69,70,71]. Persistent external and internal insults lead to excessive extracellular matrix deposition, cell death, and the distortion of systemic tissue architecture. The degree of aging is also closely related to an individual’s quality of life, their genetic makeup, and their reduced capacity for self-regeneration, which reflects the process of internal cellular decline and telomere shortening [72,73]. The early monitoring of skin health and targeted intervention for fibroblasts, collagen, and elastin, along with telomere length, are crucial steps in assessing the degree of senescence [74]. The ability of AT-MSCs to regenerate damaged tissue is primarily attributed to the autocrine and paracrine actions of their secretome, with their exosomes playing a crucial role. Exosomes derived from human AT-MSCs have been shown to stimulate the migration and proliferation of skin fibroblasts. Recent studies on MSCs and the derived exosomes used to address age-related diseases have shown significant results in lung function, diabetes, limb circulation, skin fibrosis, and skin ulcers [75].

In vivo MSC transplantation has been shown to delay senescence. Surprisingly, implanted stem cells were not directly detected in tissues and cells, suggesting their ability to act indirectly through paracrine activities and via exosomes, also detectable at the level of telomeres and telomerase [76,77,78]. This paracrine activity has been considered vital for immunomodulation, tissue repair, and regeneration [78]. Unlike MSCs, which require direct cell-to-cell contact and paracrine function, exosomes perform targeted communication functions and deliver concentrates directly to recipient cells even over long distances [69,70,71,72]. Like stem cells, exosomes possess important immunomodulatory and anti-inflammatory functions and are completely immune to rejection. They maintain the stemness and plasticity typical of pluripotent and multipotent stem cells, slow down cellular senescence, and control tumorigenesis in stem cell transplantation [79,80,81,82]. These advantages make exosomes ideal tools in anti-aging procedures and in various immune-based therapeutic strategies, showing great potential for neurodegenerative diseases and cancer [79,80,81,82].

### 9.2. The Outcomes of This Exploratory Research Study

In this study, we followed the ISEV guidelines that served as a critical foundation to ensure the quality and reproducibility of exosome research [83]. Currently, in Vietnam and Italy, although exosomes are widely discussed, only a few studies have been published on the processes of exosome isolation, purification, and application in medical treatments. Overall, although preliminary research and testing on exosomes have begun in both countries, standardized protocols and specific evaluation methods for exosome isolation remain unexplored. Therefore, this study also aimed to address the need for more detailed information on the safety, efficacy, and isolation of exosomes for application in research and medical practice.

In summary, the present study did not observe any adverse effects of AT-MSC-derived exosomes either in vitro or in in vivo experiments conducted on human fibroblasts and Albino species, supporting the safety of MSC-derived exosomes. The results reported in these studies underline the significant therapeutic and regenerative potential of exosome-based therapies, including their ability to induce protection from stressors such as H_2_O_2_ and UVB, mitigate ROS, SA-β-Gal, and inflammation, restore immune function, and enhance tissue repair. Exosomes offer a simpler structure, and non-toxic properties compared to stem cells, which have complex structures and associated safety concerns, representing an opportunity to develop new acellular treatments for various health conditions. This is a critical step in evaluating the potential therapeutic applications of exosomes, as it helps determine their biocompatibility and suitability for various biomedical applications [84].

#### Overall Fibroblast and Histological Skin Assessment After Exosome Transplantation

We characterized both AT-MSCs and their exosomes by using specific procedures, providing a reliable exosome source for basic research and clinical trials, particularly in regenerative and antiaging medicine. We assessed the size of exosome vesicles and quantified them by using the Dynamic Light Scattering (DLS) technique. The exosomes’ fluorescently labeled antibodies were stained against CD9, CD63, and CD81 after incubating the vesicles with beads during flow cytometry preparation. The results were seen under a confocal fluorescence microscope. Exosomes were extensively isolated, and their membrane proteins were quantified via the Bradford assay before being cultured in appropriate flasks [85]. The average quantification results indicated that the exosome suspension had a concentration of approximately 45.22 µg/mL (n = 15). The exosomes we sampled were also analyzed for the presence of heavy metals and their degree of toxicity.

This study was conducted in vitro and in vivo. This study is one of the few to use AT-MSC-derived exosomes to treat a mouse model exposed to UVB radiation and a cellular model exposed to H_2_O_2_. AT-MSC-derived exosomes effectively reduced the severity of UV exposure in mice by promoting extracellular matrix repair, angiogenesis, and collagen and elastin regeneration, and inhibiting inflammation.

In our experiments, senescence-associated SA-β-Gal and ROS activity levels were assessed histochemically as a standard marker for cellular senescence after exosome, H_2_O_2_, and UVB exposure. Indeed, since the early 2000s, it has been recognized that OS, determined by an uncontrolled increase in ROS, can induce both the shortening and lengthening of telomeres, depending on the severity and duration of exposure to H_2_O_2_ and UVB [86,87]. The in vitro results demonstrated that cells treated with exosomes at a concentration of 5.0 µg/mL exhibited the lowest ROS and SA-β-Gal expression levels, along with increased cell viability, greater cell density, and longer telomere length. This suggests that fibroblasts in this experimental group experienced the least damage from oxidizing agents, providing the highest protective ability and the lowest oxidative stress among the tested conditions.

Without a doubt, one of the most intriguing aspects of exosomes from a scientific point of view is the presence of miRNAs [88]. The observation that miRNAs are potential modulators of skin and dermis regeneration led us to investigate whether our exosomes could express them and whether we could observe effects on collagen and elastin content in vivo. One of the key findings of this study is the confirmation of the expression of specific miRNAs, such as miRNA-3196 and miRNA-203A and B, within our exosomes. As important post-transcriptional regulators, miRNAs play a critical role in mitigating UVB-induced skin photoaging through multiple pathways [89,90,91]. miRNA-3196 belongs to the miR-379-410 cluster, known to be highly expressed in the placenta and associated with brain, central nervous system, and circulatory development; miRNA-203A and B belong to the miR-203 family. Both miRNA 203 and miRNA 3196 are highly conserved in adipose tissue stem cells [89,90,91]. Both miRNA-203 and miRNA-3196 showed the ability to stimulate the hTERT and enhance innate and adaptive immune responses, exerting an immunomodulatory activity, suppressing proinflammatory cytokines, such as TNF-α and IL-6, and mitigating inflammation and secondary damages [90,91,92]. Although the specific mechanism remains unclear, proteins and miRNA loading could be considered major causes, as demonstrated by the disruption of these effects through RNase treatments and by the reduction in telomeres in control groups [92,93,94].

The cell cycle analysis results obtained via flow cytometry, typically presented as DNA distribution histograms of the cell population, also confirmed the enhancing and protective abilities of exosomes towards fibroblasts exposed to H_2_O_2_. The modulation of the fibroblast cell cycle by exosomes was assessed based on the proportion of cells in the G1, S, G2, and G2/G1 phases. The results indicated that, among the experimental conditions, the exosome concentration of 5.0 µg/mL yielded the highest proportion of fibroblasts in the S phase (10.32%) and G2 phase (16.21%), which are related to DNA replication and preparation for cell division and mitosis (Figure 10). These outcomes underscore the potential of exosomes in regenerative medicine and anti-aging therapies, paving the way for future research and clinical development.

We would like to point out that, although in the groups of fibroblasts subjected to senescence treatment we observed a statistically significant increase in telomere length in hF cell cultures exposed to exosomes before and after H_2_O_2_, in the control group (n.5), composed of hFs exposed only to H_2_O_2_, we observed a very slight increase in telomeres compared to the hFs of group 1 only (Figure 18). This event can be attributed to several factors, including the complex interaction between OS and telomere maintenance, the specific cellular context, and the possibility of compensatory mechanisms such as telomerase activation triggered by H_2_O_2_-induced oxidative stress, even though it was less pronounced than the effect seen in the exosome-treated groups [95].

Similarly, our in vivo findings for mouse models demonstrated that exosomes have the potential to mitigate skin damage caused by UVB radiation and confirmed statistically significant results in skin tissue, particularly in the context of wound healing and skin rejuvenation [96]. This study also sheds new light on the understanding of how the skin structure is kept and regulated. In addition to the release of soluble factors, membrane-enclosed vesicles such as exosomes—carrying membrane proteins and cytosolic components likely contribute to the maintenance of skin homeostasis. These effects included improved UVB damage closure rates, increased vascularization, increased collagen and elastin deposition, and a reduction in fibrogenesis (Figure 27 and Figure 28) (data was considered statistically significant; the *p*-value was less than a predetermined alpha (α) or 0.05) (Figure 29 and Figure 30). UV rays in sunlight, consisting of UVA (320–400 nm), UVB (280–320 nm), and UVC (200–280 nm), are a major environmental factor in causing skin photoaging and cell damage due to DNA mutations and indirectly to SO elevation [96]. Dermal fibroblasts exposed to UVB showed activate cell surface receptors, which subsequently stimulate MAPK signaling pathways, whose signal transduction is linked to transcription factor activator protein-1 (AP-1), which reduces the synthesis of type 1 collagen and increases the expression of MMPs [96,97]. When exosomes were applied to mice, we observed the increased protection and repair activity of collagen and elastin structures both before and after UVB irradiation. Histological analyses of each tissue group were performed, confirming a normalized state like that of the group not exposed to UVB, attributable to the expression of 203-A and B and 3196 mRNAs, known to play key roles in the maintenance of type I collagen and elastin. While miRNA-3196 mitigates inflammatory responses, miRNA-203 is predominantly associated with epithelial cell differentiation, maintaining epithelial cell stability and promoting tissue repair [98,99,100]. In contrast, the untreated group showed pronounced collagen and elastin damage, characterized by a severely thinner dermis. By attenuating and modulating excessive inflammatory responses, exosomes created a favorable environment for skin recovery, results that were statistically significant. Therefore, we assumed that our findings also showed the exosomes’ capacity to enhance stimulatory and protective functions through the presence of growth factors, such as TGF-β and VEGF [101,102,103]. Detailed investigations into their roles will substantially contribute to the diagnosis and treatment of complex diseases and the development of exosome-based therapies in the future [104]. Complementary techniques are necessary for a comprehensive identification and evaluation of exosomes. In studies focused on clinical applications or mechanistic insights, a rigorous sample quality control and the use of advanced methodologies are critical [105,106].

## 10. Conclusions and Final Consideration and Limitations

In conclusion, our study presents compelling evidence supporting the therapeutic potential of AT-MSC-derived exosomes in mitigating the pathogenesis and aging effects of H_2_O_2_ and UVB. They have proved their ability to reduce oxidative markers, increase dermoprotective gene expression, and improve fibroblast motility in vitro and collagen and elastin quality in ex vivo models, ultimately enhancing the repair of oxidative damage due to SA-β-Gal, increasing ROS, and supporting skin regeneration. The presence of miRNA-3196 and miRNA-203 offers profound insights into biological regulatory and regenerative mechanisms and unlocks significant potential for precision medicine. Despite the promising results, we are well aware that the study has several limitations. Larger cohorts are needed to ensure the accuracy and precision of the results, and more robust molecular insights are also a priority. Detailed investigations into their roles will substantially contribute to the diagnosis and treatment of complex diseases and the development of exosome-based therapies in the future. In addition, the use of human adipose-derived stem cell lines in the study may pose a limitation, as individual patient factors, such as advanced age, obesity, and pre-existing conditions, can negatively impact the regenerative potential of these cells and their exosomes. These factors may negatively affect the exosomes’ ability to grow and differentiate, meaning the therapeutic effects observed in the study may not apply to all patient populations.

## Figures and Tables

**Table 1:** Data and outcomes of the experimental procedure in vivo.

Experiment Performance				
Group	Number of Mice	Method	Visual Assessment	Histological Assessment
**NEGATIVE CONTROL**	Seven mice (from the beginning): - Sacrifice two mice for each 2-week period. - One mouse for backup, used in week 6.	- No exosome exposure. - No **UVB irradiation**.	Capture skin of two mice with digital camera at the time points of week 2, week 4, and week 6.	- At the time points of **week 2, week 4, and week 6**, conduct histological assessment (after visual assessment). - Histological assessment: **HE staining and Trichrome staining.** - **Two mice used at each time point.**
**POSITIVE CONTROL**	Seven mice (from the beginning): - Sacrifice two mice for each 2-week period. - One mouse for backup, used in week 6.	- No exosome exposure. - **UVB irradiation**.	Capture skin of two mice with digital camera at the time points of week 2, week 4, and week 6.	- At the time points of **week 2, week 4, and week 6**, conduct histological assessment (after visual assessment). - Histological assessment: **HE staining and Trichrome staining.** - **Two mice used at each time point.**
**EXO-50**	Seven mice (from the beginning): - Sacrifice two mice for each 2-week period. - One mouse for backup, used in week 6.	- Apply **20 μL exosomes at 50 μg/mL** on shaved dorsal skin of each mouse (2 × 2 cm). - After **3 h** from exosomes exposure, perform **UVB irradiation** on skin.	Capture skin of two mice with digital camera at the time points of week 2, week 4, and week 6.	- At the time points of **week 2, week 4, and week 6**, conduct histological assessment (after visual assessment). - Histological assessment: **HE staining and Trichrome staining.** - **Two mice used at each time point.**

**Table 2:** Skin wrinkle severity according to Agrawal criteria.

Score	Description
0	No wrinkles or sagging; small cracks aligned with the spine.
1	Appearance of small wrinkles perpendicular to the body axis.
2	Disappearance of small wrinkles.
3	Appearance of shallow wrinkles on the back perpendicular to the body axis.
4	Presence of a few shallow wrinkles with initial signs of sagging.
5	Numerous deep, permanent wrinkles.
6	Multiple deep wrinkles with tumor/skin lesion development.

**Table 6:** Viability assessment of exosomes on fibroblasts.

No.	Experimental Group	Cell Viability (%)	Mean ± SD
1	Negative Control	100.00	0.00
2	Positive Control	16.46	0.90
3	Exo-1	92.42	5.84
4	Exo-5	93.90	17.12
5	Exo-10	76.06	13.72
6	Exo-15	73.51	8.87

**Table 7:** Fibroblast cell cycle evaluation.

No.	Experimental Group	% Fibroblasts in G1 Phase	% Fibroblasts in S Phase	% Fibroblasts in G2 Phase
1	Negative Control	82.33 ± 3.05	5.34 ± 1.21	12.35 ± 4.26
2	Positive Control	83.16 ± 4.72	7.18 ± 1.51	8.01 ± 5.55
3	Exo-0.1	81.24 ± 2.91	7.93 ± 0.89	10.84 ± 2.01
4	Exo-0.5	86.34 ± 3.98	5.03 ± 1.58	8.64 ± 5.56
5	Exo-1.0	83.80 ± 0.75	6.97 ± 4.36	9.24 ± 5.11
6	Exo-5.0	73.47 ± 1.99	10.32 ± 7.14	16.21 ± 5.15

**Table 11:** Comparison of collagen fiber structure in the test area at the 2-week time point.

Group	Collagen Density	Collagen Arrangement	Epidermal Thickness	Damage Characteristics
Negative Control	Dense, intact	Well preserved and parallel to dermis	Thick dermis, no damages	Intact skin, no inflammation
Positive Control	Reduced in density, numerous breaks	Disorganized, short, fragmented	Thinned dermis due to UVB damage	Initial recovery, mild inflammation
Research (exosome-treated)	Improved density compared to positive control	Reorganized, less disordered	Thicker dermis than positive control, but below negative control	Inflammation, swelling, collagen degeneration present

**Table 12:** Comparison of collagen fiber structure of the tested area at 4 weeks.

Group	Collagen Density	Collagen Arrangement	Epidermal Thickness	Damage Characteristics
Negative Control	High, long, continuous fibers	Orderly, parallel to epidermis	Stable thickness, firm, elastic skin	Intact skin, no inflammation or damage
Positive Control	Further reduced, loosened structure	Short, severely disordered	Thin dermis, sagging skin, mild wrinkles	Persistent inflammation, reduced elasticity, small scars
Research (exosome-treated)	Recovered density, like negative control	Well organized, fewer breaks	Dermis thickness similar to negative control	No scars, good dermal recovery, lower inflammation, less wrinkling/sagging than positive control

**Table 13:** Comparison of elastin fiber structure in the test area at 2 weeks.

Group	Elastin Density	Elastin Fiber Structure	Skin Elasticity	Damage Characteristics
Negative Control	Intact, evenly distributed throughout the epithelium	Parallel, well organized	Excellent elasticity	No signs of damage, aging, or inflammation
Positive Control	Sharply reduced, numerous disruptions	Fragmented, shortened, heavily disrupted, forming abnormal clusters	Markedly reduced elasticity	Small wrinkles, loose connective tissue, mild sagging
Research (exosome-treated)	Mild recovery, higher than positive control	Longer fibers, fewer disruptions than positive control	Improved elasticity	Reduced wrinkles, firmer skin compared to positive control

**Table 14:** Comparison of elastin fiber structure in the test area at 4 weeks.

Group	Elastin Density	Elastin Fiber Structure	Skin Elasticity	Damage Characteristics
Negative Control	High, long, continuous fibers	Well organized arrangement	Good elasticity	No damage, firm skin
Positive Control	Sharply reduced rispetto negative control, slight increase from 2 weeks	Fragmented, heavily disrupted, numerous abnormal clusters	Poor elasticity, persistent sagging	More pronounced wrinkles, reduced firmness, mild sagging
Research (exosome-treated)	Significant recovery with respect to positive control	Longer fibers, fewer disruptions and abnormal clusters than positive control	Markedly improved elasticity with respect to positive control	Reduced wrinkles, firmer skin, less aging than positive control

**Table 15:** Overall histological assessment of collagen and elastin from murine models at 6 weeks.

Criterion	Negative Control	Positive Control	Research Group
Collagen Density	High	Sharply reduced	Well recovered
Collagen Structure	Well organized arrangement	Disrupted, disordered	Well recovered, minimal disruptions
Elastin Density	High	Sharply reduced	Well recovered
Elastin Structure	Intact	Fragmented, deformed	Fewer disruptions, better arrangement
Skin Elasticity	Excellent	Sharply reduced, sagging	Markedly recovered
Inflammation Status	None	Mild inflammation, fibrosis	Significantly reduced inflammation
Wrinkles	None	Pronounced	Significantly reduced
Scarring/Fibrosis	None	Small scars, fibrosis	No scars, good tissue recovery

## Data Availability

The raw data supporting the conclusions of this article will be made available by the authors on request.

## References

[1] Lee J.H., Won Y.J., Kim H., Choi M., Lee E., Ryou B., Lee S.-G., Cho B.S. (2023). Adipose Tissue-Derived Mesenchymal Stem Cell-Derived Exosomes Promote Wound Healing and Tissue Regeneration. Int. J. Mol. Sci..

[2] Dominici M., Le Blanc K., Mueller I., Slaper-Cortenbach I., Marini F., Krause D., Deans R., Keating A., Prockop D., Horwitz E. (2006). Minimal criteria for defining multipotent mesenchymal stromal cells. The International Society for Cellular Therapy position statement. Cytotherapy.

[3] Kuca-Warnawin E., Kurowska W., Plebańczyk M., Wajda A., Kornatka A., Burakowski T., Janicka I., Syrowka P., Skalska U. (2023). Basic Properties of Adipose-Derived Mesenchymal Stem Cells of Rheumatoid Arthritis and Osteoarthritis Patients. Pharmaceutics.

[4] Fiolin J., Dilogo I.H., Antarianto R.D., Pontoh L.A. (2022). Isolation and Characterization of Adipose-Derived Mesenchymal Stem Cell Exosomes: An In-Vitro Study. J. Profesi Med. J. Kedokt. Dan Kesehat..

[5] Galipeau J., Krampera M., Barrett J., Dazzi F., Deans R.J., DeBruijn J., Dominici M., Fibbe W.E., Gee A.P., Gimble J.M. (2016). International Society for Cellular Therapy perspective on immune functional assays for mesenchymal stromal cells as potency release criterion for advanced phase clinical trials. Cytotherapy.

[6] Zaripova L.N., Midgley A., Christmas S.E., Beresford M.W., Pain C., Baildam E.M., Oldershaw R.A. (2023). Mesenchymal Stem Cells in the Pathogenesis and Therapy of Autoimmune and Autoinflammatory Diseases. Int. J. Mol. Sci..

[7] Aprile D., Patrone D., Peluso G., Galderisi U. (2024). Multipotent/pluripotent stem cell populations in stromal tissues and peripheral blood: Exploring diversity, potential, and therapeutic applications. Stem Cell Res. Ther..

[8] Alfaqeh H.H., Idrus R.B.H., Saim A.B., Nordin A. (2025). Synergistic Effects of Natural Products and Mesenchymal Stem Cells in Osteoarthritis Treatment: A Narrative Review. Curr. Issues Mol. Biol..

[9] Liu Q., Li S., Dupuy A., Mai H.L., Sailliet N., Logé C., Robert J.-M.H., Brouard S. (2021). Exosomes as New Biomarkers and Drug Delivery Tools for the Prevention and Treatment of Various Diseases: Current Perspectives. Int. J. Mol. Sci..

[10] Suh J.H., Joo H.S., Hong E.B., Lee H.J., Lee J.M. (2021). Therapeutic Application of Exosomes in Inflammatory Diseases. Int. J. Mol. Sci..

[11] Yan B., Lv S., Tong P., Yan L., Chen Z., Zhou L., Yuan Q., Guo L., Shan L. (2022). Intra-Articular Injection of Adipose-Derived Stem Cells Ameliorates Pain and Cartilage Anabolism/Catabolism in Osteoarthritis: Preclinical and Clinical Evidences. Front. Pharmacol..

[12] Storti G., Foti R., Foti R., Palmesano M., Patacchiola M., Incognito D., Cervelli G., Longo B., Scioli M.G., Fiorelli E. (2025). A Comprehensive Exploration of the Biological Effects of Adipose-Derived Stem Cells in the Treatment of Systemic Sclerosis. Cells.

[13] Rosa I., Romano E., Fioretto B.S., Matucci-Cerinic M., Manetti M. (2021). Adipose-Derived Stem Cells: Pathophysiologic Implications vs Therapeutic Potential in Systemic Sclerosis. World J. Stem Cells.

[14] Rozier P., Maumus M., Bony C., Maria A.T.J., Sabatier F., Jorgensen C., Guilpain P., Noël D. (2021). Extracellular Vesicles Are More Potent than Adipose Mesenchymal Stromal Cells to Exert an Anti-Fibrotic Effect in an In Vitro Model of Systemic Sclerosis. Int. J. Mol. Sci..

[15] Zayed M.A., Sultan S., Alsaab H.O., Yousof S.M., Alrefaei G.I., Alsubhi N.H., Alkarim S., Al Ghamdi K.S., Bagabir S.A., Jana A. (2022). Stem-Cell-Based Therapy: The Celestial Weapon against Neurological Disorders. Cells.

[16] Lau C.S., Park S.Y., Ethiraj L.P., Singh P., Raj G., Quek J., Prasadh S., Choo Y., Goh B.T. (2024). Role of Adipose-Derived Mesenchymal Stem Cells in Bone Regeneration. Int. J. Mol. Sci..

[17] Fazzina R., Iudicone P., Fioravanti D., Bonanno G., Totta P., Zizzari I.G., Pierelli L. (2016). Potency testing of mesenchymal stromal cell growth expanded in human platelet lysate from different human tissues. Stem Cell Res. Ther..

[18] Choudhery M.S., Badowski M., Muise A., Pierce J., Harris D.T. (2015). Subcutaneous Adipose Tissue-Derived Stem Cell Utility Is Independent of Anatomical Harvest Site. BioResearch Open Access.

[19] López-Otín C., Blasco M.A., Partridge L., Serrano M., Kroemer G. (2013). The hallmarks of aging. Cell.

[20] Tenchov R., Sasso J.M., Wang X., Zhou Q.A. (2024). Aging Hallmarks and Progression and Age-Related Diseases: A Landscape View of Research Advancement. ACS Chem. Neurosci..

[21] Jones C.H., Dolsten M. (2024). Healthcare on the brink: Navigating the challenges of an aging society in the United States. npj Aging.

[22] Zheng L., He S., Wang H., Li J., Liu Y., Liu S. (2024). Targeting Cellular Senescence in Aging and Age-Related Diseases: Challenges, Considerations, and the Emerging Role of Senolytic and Senomorphic Therapies. Aging Dis..

[23] Semeraro M.D., Smith C., Kaiser M., Levinger I., Duque G., Gruber H.J., Herrmann M. (2020). Physical activity, a modulator of aging through effects on telomere biology. Aging.

[24] Zuo L., Prather E.R., Stetskiv M., Garrison D.E., Meade J.R., Peace T.I., Zhou T. (2019). Inflammaging and Oxidative Stress in Human Diseases: From Molecular Mechanisms to Novel Treatments. Int. J. Mol. Sci..

[25] Xi H., Li C., Ren F., Zhang H., Zhang L. (2013). Telomere, aging and age-related diseases. Aging Clin. Exp. Res..

[26] Huang X., Huang L., Lu J., Cheng L., Wu D., Li L., Zhang S., Lai X., Xu L. (2025). The relationship between telomere length and aging-related diseases. Clin. Exp. Med..

[27] Gargiulo C., Pham V.H., Nguyen K.C.D., Trieu V.L.H., Duy T.H., Abe K., Aityan S., Shiffman M. (2016). Autologous Peripheral Blood Stem Cells Increase the Telomere Length in Patient: A Case Report of 13 Patients. J. Stem Cell Res. Ther..

[28] Hayflick L. (1965). The limited in vitro lifetime of human diploid cell strains. Exp. Cell Res..

[29] Tucker L.A., Bates C.J. (2024). Telomere Length and Biological Aging: The Role of Strength Training in 4814 US Men and Women. Biology.

[30] Gao X., Yu X., Zhang C., Wang Y., Sun Y., Sun H., Zhang H., Shi Y., He X. (2022). Telomeres and Mitochondrial Metabolism: Implications for Cellular Senescence and Age-related Diseases. Stem Cell Rev. Rep..

[31] Saito Y., Yamamoto S., Chikenji T.S. (2024). Role of cellular senescence in inflammation and regeneration. Inflamm. Regen..

[32] Li C., Yuan Y., Jia Y., Zhou Q., Wang Q., Jiang X. (2025). Cellular senescence: From homeostasis to pathological implications and therapeutic strategies. Front. Immunol..

[33] Kobayashi H., Umetani M., Nishio M., Shigetomi H., Imanaka S., Hashimoto H. (2025). Molecular Mechanisms of Cellular Senescence in Age-Related Endometrial Dysfunction. Cells.

[34] Walker B.R., Moraes C.T. (2022). Nuclear-Mitochondrial Interactions. Biomolecules.

[35] Palamarchuk A.I., Kovalenko E.I., Streltsova M.A. (2023). Multiple Actions of Telomerase Reverse Transcriptase in Cell Death Regulation. Biomedicines.

[36] Assalve G., Lunetti P., Rocca M.S., Cosci I., Di Nisio A., Ferlin A., Zara V., Ferramosca A. (2025). Exploring the Link Between Telomeres and Mitochondria: Mechanisms and Implications in Different Cell Types. Int. J. Mol. Sci..

[37] Lee Y.H., Park J.Y., Lee H., Song E.S., Kuk M.U., Joo J., Oh S., Kwon H.W., Park J.T., Park S.C. (2021). Targeting Mitochondrial Metabolism as a Strategy to Treat Senescence. Cells.

[38] Stabenow L.K., Zibrova D., Ender C., Helbing D.L., Spengler K., Marx C., Wang Z.Q., Heller R. (2022). Oxidative Glucose Metabolism Promotes Senescence in Vascular Endothelial Cells. Cells.

[39] Schwartzenberg-Bar-Yoseph F., Armoni M., Karnieli E. (2004). The tumor suppressor p53 down-regulates glucose transporters GLUT1 and GLUT4 gene expression. Cancer Res..

[40] Davalli P., Mitic T., Caporali A., Lauriola A., D’Arca D. (2016). ROS, cell senescence, and novel molecular mechanisms in aging and age-related diseases. Oxidative Med. Cell. Longev..

[41] Stojanovic B., Jovanovic I., Dimitrijevic Stojanovic M., Stojanovic B.S., Kovacevic V., Radosavljevic I., Jovanovic D., Miletic Kovacevic M., Zornic N., Arsic A.A. (2025). Oxidative Stress-Driven Cellular Senescence: Mechanistic Crosstalk and Therapeutic Horizons. Antioxidants.

[42] Tarasov V., Jung P., Verdoodt B., Lodygin D., Epanchintsev A., Menssen A., Meister G., Hermeking H. (2007). Differential regulation of microRNAs by p53 revealed by massively parallel sequencing: miR-34a is a p53 target that induces apoptosis and G1-arrest. Cell Cycle.

[43] Wang H., Guo M., Wei H., Chen Y. (2023). Targeting p53 pathways: Mechanisms, structures and advances in therapy. Signal Transduct. Target. Ther..

[44] van Soest D.M.K., Polderman P.E., den Toom W.T.F., Keijer J.P., van Roosmalen M.J., Leyten T.M.F., Lehmann J., Zwakenberg S., De Henau S., van Boxtel R. (2024). Mitochondrial H_2_O_2_ release does not directly cause damage to chromosomal DNA. Nat. Commun..

[45] Lettieri-Barbato D., Aquilano K., Punziano C., Minopoli G., Faraonio R. (2022). MicroRNAs, Long Non-Coding RNAs, and Circular RNAs in the Redox Control of Cell Senescence. Antioxidants.

[46] Cruz-Ramos J.A., de la Mora-Jiménez E., Llanes-Cervantes B.A., Damián-Mejía M.A. (2025). MicroRNAs in the mitochondria-telomere axis: New insights into cancer development and potential therapeutic targets. Genes.

[47] de Almeida A.J.P.O., de Oliveira J.C.P.L., da Silva Pontes L.V., de Souza Júnior J.F., Gonçalves T.A.F., Dantas S.H., de Almeida Feitosa M.S., Silva A.O., de Medeiros I.A. (2022). ROS: Basic Concepts, Sources, Cellular Signaling, and its Implications in Aging Pathways. Oxidative Med. Cell. Longev..

[48] Kim H.J., Kim B., Byun H.J., Yu L., Nguyen T.M., Nguyen T.H., Fare P.A., Kim E.J., Cheong K.A., Kim K.S. (2021). Resolvin D1 suppresses H_2_O_2_-induced senescence in fibroblasts by inducing autophagy through the miR-1299/ARG2/ARL1 axis. Antioxidants.

[49] Yu X., Odenthal M., Fries J.W.U. (2016). Exosomes as miRNA carriers: Formation–Function–Future. Int. J. Mol. Sci..

[50] Salehi M., Kamali M.J., Arab D., Safaeian N., Ashuori Z., Maddahi M., Latifi N., Jahromi A.M. (2024). Exosomal microRNAs in regulating cancer cell resistance to apoptosis. Biochem. Biophys. Rep..

[51] Luo L., An X., Xiao Y., Sun X., Li S., Wang Y., Sun W., Yu D. (2024). Mitochondria-related microRNAs and their role in cellular senescence. Front. Physiol..

[52] Di Micco R., Krizhanovsky V., Baker D., d’Adda di Fagagna F. (2021). Cellular senescence in ageing: From mechanisms to therapeutic opportunities. Nat. Rev. Mol. Cell Biol..

[53] Tchou W.W., Rom W.N., Tchou-Wong K.M. (1996). Novel form of p21(WAF1/CIP1/SDI1) protein in phorbol ester-induced G2/M arrest. J. Biol. Chem..

[54] Witkiewicz A.K., Knudsen K.E., Dicker A.P., Knudsen E.S. (2011). The meaning of p16(ink4a) expression in tumors: Functional significance, clinical associations and future developments. Cell Cycle.

[55] Kumar R., Gullapalli R.R. (2024). High Throughput Screening Assessment of Reactive Oxygen Species (ROS) Generation using Dihydroethidium (DHE) Fluorescence Dye. J. Vis. Exp. JoVE.

[56] Kim H., Xue X. (2020). Detection of Total Reactive Oxygen Species in Adherent Cells by 2′,7′-Dichlorodihydrofluorescein Diacetate Staining. J. Vis. Exp. JoVE.

[57] Athar M., Chaudhury N.K., Hussain M.E., Varshney R. (2011). Hoechst 33342 induced reactive oxygen species and impaired expression of cytochrome c oxidase subunit 1 leading to cell death in irradiated human cancer cells. Mol. Cell. Biochem..

[58] Waheed T.O., Hahn O., Sridharan K., Mörke C., Kamp G., Peters K. (2022). Oxidative Stress Response in Adipose Tissue-Derived Mesenchymal Stem/Stromal Cells. Int. J. Mol. Sci..

[59] Purschke M., Rubio N., Held K.D., Redmond R.W. (2010). Phototoxicity of Hoechst 33342 in time-lapse fluorescence microscopy. Photochem. Photobiol. Sci..

[60] Soroko S.S., Skamnitskiy D.V., Gorshkova E.N., Kutova O.M., Seriev I.R., Maslennikova A.V., Guryev E.L., Gudkov S.V., Vodeneev V.A., Balalaeva I.V. (2024). The Dose Rate of Corpuscular Ionizing Radiation Strongly Influences the Severity of DNA Damage, Cell Cycle Progression and Cellular Senescence in Human Epidermoid Carcinoma Cells. Curr. Issues Mol. Biol..

[61] Yin P., Sun D., Deng Y., Zhu X., Wang Y., Yang J., Feng X. (2024). Metal-organic cage as a theranostic nanoplatform for magnetic resonance imaging guided chemodynamic therapy. Theranostics.

[62] Fuchs H., Jahn K., Hu X., Meister R., Binter M., Framme C. (2023). Breaking a Dogma: High–Throughput Live–Cell Imaging in Real–Time with Hoechst 33342. Adv. Healthc. Mater..

[63] Athar M., Chaudhury N.K., Hussain M.E., Varshney R. (2010). Hoechst 33342 induces radiosensitization in malignant glioma cells via increase in mitochondrial reactive oxygen species. Free Radic. Res..

[64] Zhang X., Gao Y., Zhang S., Wang Y., Du Y., Hao S., Ni T. (2025). The Regulation of Cellular Senescence in Cancer. Biomolecules.

[65] Jomova K., Raptova R., Alomar S.Y., Alwasel S.H., Nepovimova E., Kuca K., Valko M. (2023). Reactive oxygen species, toxicity, oxidative stress, and antioxidants: Chronic diseases and aging. Arch. Toxicol..

[66] (2009). Biological Evaluation of Medical Devices—Part 5: Tests for In Vitro Cytotoxicity.

[67] (2021). Biological Evaluation of Medical Devices—Part 10: Tests for Skin Sensitization.

[68] Navarro C., Salazar J., Díaz M.P., Chacin M., Santeliz R., Vera I., D’Marco L., Parra H., Bernal M.C., Castro A. (2023). Intrinsic and environmental basis of aging: A narrative review. Heliyon.

[69] Jin G.H., Liu Y., Jin S.Z., Liu X.D., Liu S.Z. (2007). UVB induced oxidative stress in human keratinocytes and protective effect of antioxidant agents. Radiat. Environ. Biophys..

[70] Ansary T.M., Hossain M.R., Kamiya K., Komine M., Ohtsuki M. (2021). Inflammatory Molecules Associated with Ultraviolet Radiation-Mediated Skin Aging. Int. J. Mol. Sci..

[71] Nan L., Guo P., Hui W., Xia F., Yi C. (2025). Recent advances in dermal fibroblast senescence and skin aging: Unraveling mechanisms and pioneering therapeutic strategies. Front. Pharmacol..

[72] Ho C.Y., Dreesen O. (2021). Faces of cellular senescence in skin aging. Mech. Aging Dev..

[73] Kopp W. (2024). Aging and “Age-Related” Diseases—What Is the Relation?. Aging Dis..

[74] Naharro-Rodriguez J., Bacci S., Hernandez-Bule M.L., Perez-Gonzalez A., Fernandez-Guarino M. (2025). Decoding Skin Aging: A Review of Mechanisms, Markers, and Modern Therapies. Cosmetics.

[75] Putri P.H.L., Alamudi S.H., Dong X., Fu Y. (2025). Extracellular vesicles in age-related diseases: Disease pathogenesis, intervention, and biomarker. Stem Cell Res. Ther..

[76] Lyamina S., Baranovskii D., Kozhevnikova E., Ivanova T., Kalish S., Sadekov T., Klabukov I., Maev I., Govorun V. (2023). Mesenchymal Stromal Cells as a Driver of Inflammaging. Int. J. Mol. Sci..

[77] Oveili E., Vafaei S., Bazavar H., Eslami Y., Mamaghanizadeh E., Yasamineh S., Gholizadeh O. (2023). The potential use of mesenchymal stem cells-derived exosomes as microRNAs delivery systems in different diseases. Cell Commun. Signal..

[78] Odehnalová N., Šandriková V., Hromadka R., Skaličková M., Dytrych P., Hoskovec D., Kejík Z., Hajduch J., Vellieux F., Vašáková M.K. (2025). The potential of exosomes in regenerative medicine and in the diagnosis and therapies of neurodegenerative diseases and cancer. Front. Med..

[79] Yang Y.-P., Nicol C.J.B., Chiang M.-C. (2025). A Review of the Neuroprotective Properties of Exosomes Derived from Stem Cells and Exosome-Coated Nanoparticles for Treating Neurodegenerative Diseases and Stroke. Int. J. Mol. Sci..

[80] Youssef E., Palmer D., Fletcher B., Vaughn R. (2025). Exosomes in Precision Oncology and Beyond: From Bench to Bedside in Diagnostics and Therapeutics. Cancers.

[81] Keshtkar S., Asvar Z., Najafi H., Heidari M., Kaviani M., Sarvestani F.S., Tamaddon A.M., Sadati M.S., Hamidizadeh N., Azarpira N. (2025). Exosomes as natural vectors for therapeutic delivery of bioactive compounds in skin diseases. Front. Pharmacol..

[82] Saha P., Datta S., Ghosh S., Samanta A., Ghosh P., Sinha D. (2021). Bioengineering of Extracellular Vesicles: Exosome-Based Next-Generation Therapeutic Strategy in Cancer. Bioengineering.

[83] Théry C., Witwer K.W., Aikawa E., Alcaraz M.J., Anderson J.D., Andriantsitohaina R., Antoniou A., Arab T., Archer F., Atkin-Smith G.K. (2018). Minimal information for studies of extracellular vesicles 2018 (MISEV2018): A position statement of the International Society for Extracellular Vesicles and update of the MISEV2014 guidelines. J. Extracell. Vesicles.

[84] Gorshkov A., Purvinsh L., Brodskaia A., Vasin A. (2022). Exosomes as Natural Nanocarriers for RNA-Based Therapy and Prophylaxis. Nanomaterials.

[85] Kielkopf C.L., Bauer W., Urbatsch I.L. (2020). Bradford Assay for Determining Protein Concentration. Cold Spring Harb. Protoc..

[86] von Zglinicki T. (2002). Oxidative stress shortens telomeres. Trends Biochem. Sci..

[87] Purnianto A., Mawal C., Kulkarni M.M., Su H., Koukoulis T.F., Wongsodirdjo P., Hung Y.H., Ayton S., Bush A.I., Barnham K.J. (2024). Small extracellular vesicles contain metals and transfer metal intercellularly. J. Extracell. Biol..

[88] Tienda-Vázquez M.A., Hanel J.M., Márquez-Arteaga E.M., Salgado-Álvarez A.P., Scheckhuber C.Q., Alanis-Gómez J.R., Espinoza-Silva J.I., Ramos-Kuri M., Hernández-Rosas F., Melchor-Martínez E.M. (2023). Exosomes: A Promising Strategy for Repair, Regeneration and Treatment of Skin Disorders. Cells.

[89] Wang W., Yin J. (2025). Exosomal miR-203 from bone marrow stem cells targets the SOCS3/NF-κB pathway to regulate neuroinflammation in temporal lobe epilepsy. World J. Stem Cells.

[90] Giammona A., Di Franco S., Lo Dico A., Stassi G. (2024). The miRNA Contribution in Adipocyte Maturation. Non-Coding RNA.

[91] Song S., Johnson K.S., Lujan H., Pradhan S.H., Sayes C.M., Taube J.H. (2021). Nanoliposomal Delivery of MicroRNA-203 Suppresses Migration of Triple-Negative Breast Cancer through Distinct Target Suppression. Non-Coding RNA.

[92] Giunco S., Petrara M.R., Indraccolo S., Ciminale V., De Rossi A. (2025). Beyond Telomeres: Unveiling the Extratelomeric Functions of TERT in B-Cell Malignancies. Cancers.

[93] Tapparo M., Pomatto M.A.C., Deregibus M.C., Papadimitriou E., Cavallari C., D’Antico S., Collino F., Camussi G. (2021). Serum Derived Extracellular Vesicles Mediated Delivery of Synthetic miRNAs in Human Endothelial Cells. Front. Mol. Biosci..

[94] Li S., Liu F., Zhang S., Sun X., Li X., Yue Q., Su L., Yang S., Zhao L. (2025). Lavender Exosome-Like nanoparticles attenuate UVB-Induced Photoaging via miR166-Mediated inflammation and collagen regulation. Sci. Rep..

[95] Haendeler J., Hoffmann J., Brandes R.P., Zeiher A.M., Dimmeler S. (2003). Hydrogen peroxide triggers nuclear export of telomerase reverse transcriptase via Src kinase family-dependent phosphorylation of tyrosine 707. Mol. Cell. Biol..

[96] Sil A., Chakraborty D. (2024). miRNA: The Next Frontier in Dermatology Research and Therapeutics. Indian J. Dermatol..

[97] Cavinato M., Jansen-Dürr P. (2017). Molecular mechanisms of UVB-induced senescence of dermal fibroblasts and its relevance for photoaging of the human skin. Exp. Gerontol..

[98] Li X., Ponandai-Srinivasan S., Nandakumar K.S., Fabre S., Xu Landén N., Mavon A., Khmaladze I. (2021). Targeting microRNA for improved skin health. Health Sci. Rep..

[99] Lena A.M., Shalom-Feuerstein R., Rivetti di Val Cervo P., Aberdam D., Knight R.A., Melino G., Candi E. (2008). miR-203 represses ‘stemness’ by repressing DeltaNp63. Cell Death Differ..

[100] McKenna D.J., McDade S.S., Patel D., McCance D.J. (2010). MicroRNA 203 expression in keratinocytes is dependent on regulation of p53 levels by E6. J. Virol..

[101] Zhang S., Yang Y., Lv X., Zhou X., Zhao W., Meng L., Zhu S., Zhang Z., Wang Y. (2024). Exosome Cargo in Neurodegenerative Diseases: Leveraging Their Intercellular Communication Capabilities for Biomarker Discovery and Therapeutic Delivery. Brain Sci..

[102] Prasai A., Jay J.W., Jupiter D., Wolf S.E., El Ayadi A. (2022). Role of Exosomes in Dermal Wound Healing: A Systematic Review. J. Investig. Dermatol..

[103] Al Halawani A., Mithieux S.M., Yeo G.C., Hosseini-Beheshti E., Weiss A.S. (2022). Extracellular Vesicles: Interplay with the Extracellular Matrix and Modulated Cell Responses. Int. J. Mol. Sci..

[104] Lohajová Behulová R., Bugalová A., Bugala J., Struhárňanská E., Šafranek M., Juráš I. (2023). Circulating exosomal miRNAs as a promising diagnostic biomarker in cancer. Physiol. Res..

[105] Bai L., Yu L., Ran M., Zhong X., Sun M., Xu M., Wang Y., Yan X., Lee R.J., Tang Y. (2025). Harnessing the Potential of Exosomes in Therapeutic Interventions for Brain Disorders. Int. J. Mol. Sci..

[106] Dilsiz N. (2024). A comprehensive review on recent advances in exosome isolation and characterization: Toward clinical applications. Transl. Oncol..

